# A systematic review of the association of diet quality with the mental health of university students: implications in health education practice

**DOI:** 10.1093/her/cyac035

**Published:** 2022-11-28

**Authors:** Solomis Solomou, Jennifer Logue, Siobhan Reilly, Guillermo Perez-Algorta

**Affiliations:** Faculty of Health and Medicine, Lancaster University, Bailrigg, Lancaster LA1 4YW, UK; Faculty of Health and Medicine, Lancaster University, Bailrigg, Lancaster LA1 4YW, UK; Faculty of Health and Medicine, Lancaster University, Bailrigg, Lancaster LA1 4YW, UK; Faculty of Health Studies, University of Bradford, Richmond Road, Bradford, West Yorkshire BD7 1DP, UK; Faculty of Health and Medicine, Lancaster University, Bailrigg, Lancaster LA1 4YW, UK

## Abstract

University students are at risk of experiencing mental health problems during the transition from home to university. This transition can also adversely affect their diet quality. This review aims to examine bidirectional associations from observational studies regarding the influence of diet quality on the mental health of university students, and vice versa. The databases PubMed, CINAHL, EMBASE, PsycINFO, The Cochrane Library and Web of Science were searched using relevant search terms. The searches were last updated on 15 July 2022. Majority of studies (36 out of 45) found that good diet quality of students was associated with better mental health in terms of depression, anxiety, stress and overall general mental well-being. Moreover, majority of studies (19 out of 23) found that stress and anxiety of students were associated with poorer diet quality. The effect sizes observed were generally small–moderate. Healthy diets of students have been associated with better mental health in terms of depression, anxiety, stress or other mental health issues. Stress experienced by university students has been associated with unhealthy diets. There are implications for health education research, as interventions to improve diet quality at the university level could reduce mental health issues; additionally, interventions to support students under stress may lead to healthier dietary habits when living on campuses. Randomized controlled trials and intervention studies are needed to further investigate these implications.

## Introduction

The transition to university from home can have both positive and negative effects. Positive effects include developing independence, lifelong friendships and networking. However, when the outcome of the life transition is positive (such as gaining new skills and paid employment), this experience can still be unpleasant and associated with life dissatisfaction [1]. Some examples of challenges refer to academic, social, personal-emotional and institutional adjustments [2]. Change is often a cause of uncertainty, which in turn can induce higher levels of stress and anxiety [3].

The transition to university from home may affect mood and overall mental health. This is a period of increased risk of onset of mental health problems [4], particularly for the onset of depression and anxiety [5].

In the United States, it is estimated that up to 50% of the students living on university campuses can be affected by mental health problems [6]. This observation appears to be an international issue. A meta-analysis of 34 international studies with university students of various years between 1990 and 2010 showed an average prevalence of depression of 30.6% [7]. Rates were substantially higher than those found in the general population (∼11%) [8]. However, there are studies that have not detected differences in the mental health of students and non-students [9]. The differences in the findings examining mental health prevalence statistics could be attributed to the various methods used by studies, as ways in which symptoms have been assessed are not always appropriate for establishing prevalence *per se*.

Definitions of mental health may also vary. The World Health Organization defines mental health as ‘our emotional, psychological, and social well-being’ [10]. The terms mental health and mental illness may be used interchangeably, however a person may experience poor mental health even without being diagnosed with a mental illness [11]. For this reason, in order to better understand the various dimensions of mental health, it may be appropriate to look for a broad range of studies that use constructs assessing for ‘mental health’; these may vary from specific symptom measures, to well-being measures, to positive self-concept, as well as include constructs such as resilience and self-concept.

The transition to university from home also appears to affect the diet quality of students, as this period is characterized by students adopting poor quality diets [12].

A meta-analysis of studies conducted in the United States, Canada, United Kingdom and Belgium showed a student weight gain of 1.4 kg over two terms [12]. This increment in weight is five times higher than the weight gain expected in the general population over a period of 1 year. In the United States, this has been observed in at least two-thirds of students during their 1st year of university [13]. These changes in diet quality are characterized by increments in the consumption of fast food, relying more on take-out food and less on fresh food [14, 15].

Organizations, such as the Food Standards Agency in the United Kingdom, have issued guidelines as to what a good quality diet should consist of [16]. For example, these guidelines include advice about the intake of fruit (≥2 servings/day), vegetables (≥3 servings/day), oily fish (≥200 g/week), fat (≤85 g/day) and non-milk extrinsic sugars (≤60 g/day) [17]. There are also specific types of diets that are in line with the above recommendations, such as the Mediterranean diet which consists of fruits, vegetables, whole grains, seafood, beans and nuts, and whose health benefits have been reported in previous literature [18]. Various diet quality measures have been devised to capture the quality of a diet according to guidelines such as the ones mentioned earlier, although studies often use food frequency questionnaires without considering a diet quality instrument.

Although not the only factor, poor quality diet has been considered a risk factor of mental health problems [19], and some argue that mental health issues could impact diet quality too [20]. Understanding this relationship could have important implications in health education practice. Current practices for addressing the mental health of university students include counselling [21], as well as cognitive, behavioural and mindfulness interventions [22]. More recently, the above interventions are being available via the internet in addition to sessions in person by university counsellors [22, 23]. Educating students about mental health has also been shown to be effective [24]. In some cases, students utilize pharmacological options, such as antidepressants prescribed by general practitioners or psychiatrists [25]. However, pharmacological and psychological interventions may not always be able to prevent or resolve mental health issues; hence, diet could be a potential target for the prevention and adjunct treatment of anxiety and depression of students [26].

Even though there is a scarcity of studies investigating the association of diet quality with mood in university students, relevant studies involving the general population are more abundant. These studies have mainly focussed on the effects of diet on depression. Reviews of cross-sectional studies have shown inverse associations of small–moderate effect size between diet quality scores and depressive symptoms [19, 27]. Moreover, a recent meta-analysis of randomized controlled trials examined the efficacy of dietary interventions for symptoms of depression in both clinical and non-clinical populations [26]. This review showed evidence that dietary interventions had a small–moderate effect on improvement of depressive symptoms. Examples of dietary interventions that were used included individualized dietary counselling, group dietary classes and standardized dietary prescription. In view of the above evidence, there is scope to understand the influence of diet on mental health of students and vice versa by performing a systematic literature review of relevant observational studies. Moreover, establishing a link between diet quality and mental health may be used for practical support involving interventions that could improve both diet quality and mental health of students.

### Review aims

To better understand the associations between diet and mental health, this review aimed to interpret study findings in the context of the diathesis–stress model [28]. Stress–diathesis models are models that can facilitate our understanding of how predispositional factors from various domains can cause susceptibility to psychopathology and eventually lead to conditions that are sufficient for the development of a mental health disorder [29]. These models may encompass multiple factors contributing to psychopathology, including biological vulnerabilities, psychological susceptibilities, social variables, environmental variables and developmental experiences [29].

The review aimed to interpret findings while taking into consideration the various risk factors that can affect students with a biological, psychological or social vulnerability to mental illness or to bad diet quality. This was in line with both the stress–diathesis model and the biopsychosocial model of health and illness [30, 31]. Biopsychosocial factors linked to mental health include stress, stressful life events, body image, physical activity, sleep, social support, use of alcohol or illicit drugs. There are also biopsychosocial factors linked to diet, such as availability and access of pre-prepared meals/fast foods on campus, lack of cooking skills, lack of culinary and basic nutritional knowledge, no previous hands-on involvement in food preparation in the family environment, limited resources including money for shopping, no easy access to healthy food and lack of companionship during meal times [32]. Hence, we aimed to look for moderators or mediators of the association between diet and mental health, in recognition that mental health and diet quality difficulties are multifaceted and underpinned by complex biopsychosocial processes.

Scoping searches did not identify any previous systematic literature reviews appraising both directions of the influence of diet quality on the mental health of university students, and vice versa. However, one previous systematic review appraising the association of mental health with the diet quality of students [20] was identified, which was published in 2018; further scoping searches showed that at least eight relevant studies were published since 2018. In 2021, another systematic review appraised the opposite direction of the association, i.e. the influence of diet on the mental health of students [33]; further scoping searches showed that at least six relevant studies were published since the data search of this review was done. None of these reviews assessed both directions of the association between diet quality and mental health. Even though studies have been treating associations with diet and mental health, and associations with mental health and diet as separate, in reality, most research cannot establish whether one is predicting the other, as they are associations. In view of this, we feel that a full picture can only be obtained by including studies in the review that have assessed either direction of the association.

Hence, the current review aims to provide knowledge by appraising studies investigating the influence of diet quality on the mental health of university students, and vice versa. This is important as the findings may have implications in health education practice. The review aims to appraise studies that have been assessed by the previous reviews [20, 33], as well as studies that have never been appraised before.

Given the fact that scoping searches indicated the majority of studies to be cross-sectional, the review did not aim to answer the question of causation in regard to the relationship between diet and mental health.

## Methods

This systematic review followed the Preferred Reporting Items for Systematic reviews and Meta-Analyses statement [34] and was registered in the PROSPERO International Prospective Register of Systematic Reviews (number CRD42020196336 at www.crd.york.ac.uk/PROSPERO). There were no discrepancies between the initial protocol and the processes that were followed.

### Search strategy

A search of the literature was performed on 1 July 2020 (date range for searches was from inception to 1 July 2020). The searches were re-run on 15 July 2022 in order to update the review with recent studies. The databases PubMed, CINAHL, EMBASE, PsycINFO, The Cochrane Library and Web of Science were searched by using the following search terms:

Student* AND (Diet* OR Nutrition OR Eat* OR Food OR Weight gain) AND (Mood OR Depress* OR Anxiety OR Stress OR Mental health).

‘Weight loss’ was not included in search terms as previous studies have reported a weight gain (rather than weight loss) in students transitioning to university. Moreover, weight loss due to depression would be more relevant to lack of appetite, which is not the focus of this review.

Both medical subject headings and free-text terms were incorporated, which were adapted according to the database searched. Google Scholar, OpenGrey and ResearchGate were also searched in order to identify any relevant grey literature. This strategy was followed in order to ensure a broad coverage of studies. The reference lists of the included studies and reviews were hand searched in order to identify any additional papers of relevance. Where further information was required, authors of retrieved studies were contacted.

### Inclusion and exclusion criteria

The review included studies published in peer-reviewed journals or grey literature, including cross-sectional and longitudinal studies, as well as review studies of observational evidence. Scoping searches did not identify any relevant intervention studies, randomized controlled trials or qualitative studies. Intervention studies examining only individual foods/nutrients or focussing only on a single food component were not considered eligible, as the focus is on whole of diet associations and effects. Hence, the focus was on observational studies, as the review aimed to gain insight into the associations of diet quality with the mental health (and vice versa) of university students in their natural environment without any external interventions.

Publication languages included English, Greek and Spanish (as these are the authors’ languages). There were no publication period restrictions.

Studies were included if they involved participants who were university students of any ethnic origin, gender and age, studying in any country, with or without a mental health diagnosis (such as depression and/or anxiety).

Studies were excluded if they involved participants who were not university students, if they studied associations of mental health with single macro/micronutrients rather than overall diet quality, or if they studied associations of mental health with nutritional supplements rather than diet quality. Studies were also excluded if they focussed on disordered eating behaviours rather than on diet quality.

### Main outcomes

The main outcomes included depression or anxiety or depressive/anxiety symptoms or other mental health symptoms (assessed by relevant scales, or as experienced subjectively by participants, or as diagnosed by health professionals) and diet quality. The review included studies using diet measures (such as food frequency questionnaires) and/or diet quality scores obtained from diet quality instruments.

### Screening

**Fig. 1.**
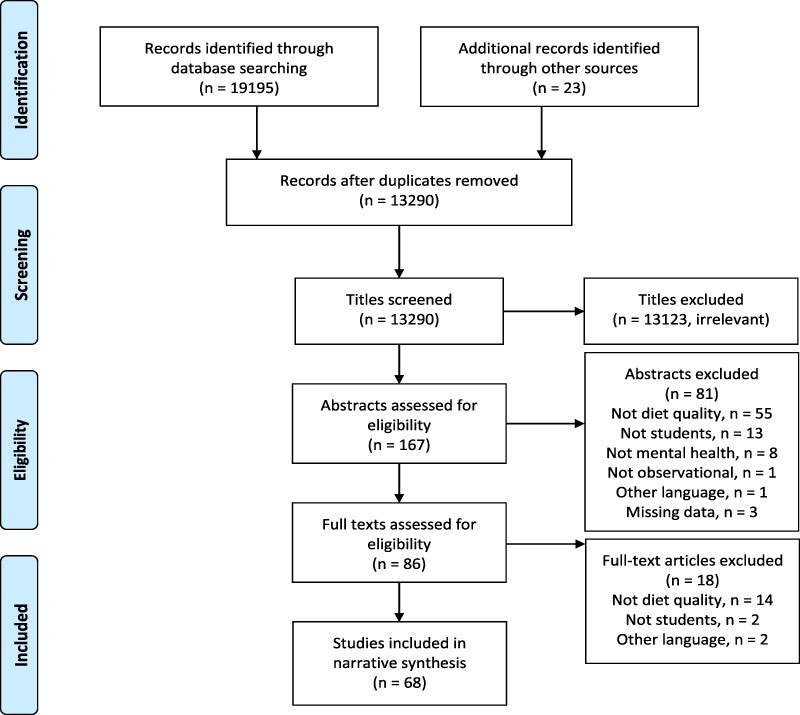
Flowchart of selected studies.

Titles were screened by author S.S. for inclusion, followed by screening of abstract and then content. Full texts were obtained in cases where abstract eligibility was considered uncertain or if title eligibility was considered uncertain and abstracts were not available. Studies were included at the abstract screening stage if abstracts were in English, Spanish or Greek, but studies were excluded if their full texts were in other language. Authors of studies were contacted when there was not enough information to decide whether a study met the inclusion criteria. All screening steps were discussed with author G.P.A. The studies meeting the inclusion criteria were selected for the review as summarized in Fig. 1.

### Data extraction

Data were extracted from observational studies by using the relevant sections of the Cochrane good practice data extraction form. Data were extracted from reviews by using a modified version of the National Institute for health and Care Excellence extraction form [35]. The data were extracted in an electronic format in order to achieve effective time management and reduce any errors during data entry.

The extracted data included the following: authors, year of publication, setting, study design, sample size, geographical location, follow-up time (if applicable), demographic and clinical characteristics of participants, measures used (where applicable) and main findings (dietary assessment tool used and score used, assessment of depression and/or anxiety, depressive and anxiety symptoms scale and threshold used), confounders used and relevant statistics. In cases where various analyses were completed, the analysis that had taken the largest number of confounders into consideration was used.

### Risk of bias/quality assessment

The quality of studies was scored into high, medium and low quality by using the Newcastle–Ottawa Quality Assessment Scale (adapted for cross-sectional studies) [36]. This instrument has a highest score of 10, with 5 points being allocated to selection (representativeness of the sample, sample size, non-respondents and ascertainment of the exposure), 2 points being allocated to comparability and 3 points being allocated to outcome (including assessment of outcome and statistical tests). The guidance of the Centre for Reviews and Dissemination [37] was used for appraising the quality of review papers. Where appropriate, discussion between the authors was used to resolve any uncertainties.

### Strategy for data synthesis

A narrative synthesis review [38] of observational studies (and of reviews of observational studies) reporting associations of diet quality with mood and mental health of university students (with or without an established mental health diagnosis), and vice versa, was performed. This was considered to be the best approach to analyse the observational data available.

## Results

Following title screening of 13 290 articles, 167 abstracts were read in full and assessed against the inclusion and exclusion criteria. Eighty-six full-text articles that met the inclusion criteria were then retrieved, and the full texts were subsequently screened against the criteria. Authors of three papers were contacted to obtain further information [39–41]. The final number of papers that were included in the review was 68 (Fig. 1).

Of the included studies, 44 primary studies investigated the influence of diet on mental health (of which 43 were cross-sectional and one was longitudinal). There was also one review identified that investigated this direction of the association. In terms of study quality (as measured by the Newcastle–Ottawa Quality Assessment Scale and the guidance of the Centre for Reviews and Dissemination), 15 studies were evaluated as of high quality, 29 were of medium quality and one was of low quality (Online Supplemental Materials 1–3). There were no relevant randomized controlled trials, intervention studies or qualitative studies identified.

In terms of studies investigating the influence of mental health on diet, one systematic review and 22 primary studies were evaluated (of which 18 were cross-sectional and four were longitudinal). Three studies were considered of high quality, 19 studies were of medium quality and one study was of low quality (Online Supplemental Materials 1–3). The inclusion criteria were not met by any relevant randomized controlled trials, intervention studies or qualitative studies.

Where diet quality instruments were used, the most common measure was the Healthy Eating Index (HEI). HEI is a measure of diet quality that assesses how well food intake aligns with key recommendations of the Dietary Guidelines for Americans [42]. In terms of mental health instruments, the most frequently used instrument was the Depression, Anxiety and Stress Scale (DASS-21), which is a set of three self-report scales designed to measure the emotional states of depression, anxiety and stress [43].

### Diet quality associations with mental health

In regard to geographical settings of studies investigating the influence of diet quality on mental health, 38% of the studies took place in Europe, 31% in the United States and Canada, 12% in Asia, 12% in the Middle East, 5% in Latin America and 2% in Africa. The studies also varied in terms of the number of study participants, from 36 to 68 559 (Table I).

**Table I.** Baseline characteristics (for studies investigating association of diet quality with mental health parameters)

Author, year	Mental health parameter	Design	Country	Age details	*N* students, sex
Açik and Cakiroglu, 2019 [44]	Depression	Cross-sectional	Turkey	Aged 19–24 years	*N* = 134 students, All females
Jeffers et al., 2019 [45]	Depression	EMA	United States	Mean age = 21 years	*N* = 30, Females: *n* = 15, Males: *n* = 15
Faghih et al., 2020 [46]	Depression, anxiety, stress, general mental well-being	Cross-sectional	Iran	Mean age = 21.5 years	*N* = 274, Females: *n* = 238, Males: *n *= 36
Ramón-Arbués et al., 2019 [64]	Depression, anxiety, stress	Cross-sectional	Spain	Mean age = 21.74 years	*N* = 1055, Females: *n* = 311, Males: *n* = 744
Abramson, 2017 [119]	Depression	Cross-sectional	United States	Age range = 18–31 years	*N* = 36, Females: *n* = 22, Males: *n* = 14
Quehl et al., 2017 [48]	Depression	Cross-sectional	Canada	Mean age = 19.1 years	*N* = 141, All females
Sakai et al., 2017 [47]	Depression	Cross-sectional	Japan	Mean age = 18 years	*N* = 3963, All females
Hamazaki et al., 2015 [50]	Depression	Cross-sectional	Japan	Mean age = 20.5 years	*N* = 4190, Females: *n* = 2066, Males: *n *= 2124
Liu et al., 2007 [51]	Depression, stress	Cross-sectional	China	Mean age = 20.4 years	*N* = 2579, Females: *n* = 1086, Males: *n *= 1493
Peltzer and Pengpid, 2017a [52]	Depression, general mental well-being, PTSD	Cross-sectional	Various	Mean age = 20.5 years	*N* = 3357, Females: *n* = 2112, Males: *n* = 1245
Peltzer and Pengpid, 2017b [53]	Depression	Cross-sectional	Various	Mean age = 20.9 years	*N* = 18 522, Females: *n* = 10 708, Males: *n* = 7758
Smith-Marek et al., 2016 [54]	Depression, PTSD	Cross-sectional	United States	89% were between the age of 18 and 21	*N* = 321, Females: *n* = 245, Males: *n* = 76
Breiholz, 2010 [120]	Depression	Cross-sectional	United States	Age not stated	*N* = 188, sex not stated
El Ansari et al., 2014 [55]	Depression, stress	Cross-sectional	United Kingdom	Mean age = 24.9 years	*N* = 3.706, Females: *n* = 2699, Males: *n* = 765, Other: *n* = 242
Mikolajczyk et al., 2009 [59]	Depression, stress	Cross-sectional	Various	Mean age = 20.6 years	*N* = 1839, Females: *n *= 1200, Males: *n* = 639
Oleszko et al., 2019 [56]	Depression	Cross-sectional	Poland	Age not stated	*N* = 959, Females: *n* = 576, Males: *n *= 383
Romijn, 2020 [57]	Depression, anxiety	Cross-sectional	United Kingdom	Mean age = 18 years	*N* = 280, Females: *n* = 231, Males: *n* = 49
Rossa-Rocor, 2019 [60]	Depression, anxiety, general mental well-being	Cross-sectional (thesis)	Canada	Mean age = 19.5 years	*N* = 339, Females: *n* = 224, Males: *n* = 109, Other: *n* = 6
Jaalouk et al., 2019 [121]	Depression	Cross-sectional	Lebanon	Mean age = 21.3 years	*N* = 457, Females: *n* = 170, Males: *n* = 287
Tran et al., 2017 [61]	Depression, anxiety	Cross-sectional	France	69% were less than 20 years old	*N* = 4184, Females: *n* = 2403, Males: *n* = 1781
Wattick et al., 2018 [58]	Depression, anxiety	Cross-sectional	United States	59.4% aged 19–21 years	*N* = 1956, Females: = 1320, Males: *n* = 636
Rossa-Rocor et al., 2021 [49]	Depression, anxiety, QoL	Cross-sectional	Canada	Mean age 19.5 years, SD 1.9	*N* = 339 students, *n* = 224 females
Stanton et al., 2021 [62]	Depression, anxiety, stress	Cross-sectional	Australia	18–24 years (*n* = 183), 25–34 years (*n* = 159), ≥ 35 (*n* = 158)	*N* = 500 students, *n* = 472 females
Attlee et al., 2022 [65]	Depression, anxiety, stress	Cross-sectional	UAE	Mean age 20.3 years, SD 1.8	*N* = 260, all females
Lee et al., 2022 [63]	Depression, anxiety, stress	Cross-sectional	Canada	88.4% were 18–24 years	*N* = 146, *n* = 127 females
Saharkhiz et al., 2021 [68]	Depression, anxiety, stress	Cross-sectional	Iran	Mean age 20.7 years, SD 2.2	*N* = 181, all females
Fabian et al., 2013 [122]	Stress	Cross-sectional	Puerto-Rico	Aged 21–30 years	*N* = 252, Females: *n* = 170, Males: *n* = 82
El Ansari et al., 2015a [66]	Stress	Cross-sectional	Finland	Median age = 21 years	*N* = 1076, Females: *n *= 762, Males: *n* = 314
Lockhart, 2017 [123]	Stress	Secondary data analysis (thesis)	United States	Mean age = 21.04 years	*N* = 68 559, Females: *n* = 44 403, Males: *n* = 23 517, Unknown: *n* = 639
Alfreeh et al., 2020 [67]	Stress	Cross-sectional	Saudi Arabia	Age range 19–35 years	*N* = 401, all females
Lo Moro et al., 2021 [71]	Mental well-being	Cross-sectional	Italy	Median age 23 years	*N* = 502, 76% females
Aceijas et al., 2017 [39]	General mental well-being	Cross-sectional	United Kingdom	Mean age = 23.6 years	*N* = 468, Females: *n* = 328, Males: *n* = 140
El Ansari et al., 2015b [69]	General mental well-being	Cross-sectional	Finland	Median age = 21 years	*N* = 1027, Males: *n* = 302, Females: *n* = 725
Hendy, 2012 [70]	General mental well-being	Longitudinal	United States	Mean age = 24.4 years	*N* = 44, Females: *n* = 33, Males: *n = *11
Lopez-Olivares, 2020 [72]	General mental well-being	Cross-sectional	Spain	Mean age = 20.97 years	*N* = 272, Females: *n* = 176, Males: *n* = 96
Mochimasu et al., 2016 [73]	General mental well-being	Cross-sectional	Japan	Mean age = 18.78 years	*N* = 62, All females
Knowlden et al., 2016 [74]	General mental well-being	Cross-sectional	United States	67% were 19–20 years	*N* = 195, Females: *n* = 138, Males: *n* = 57
Lesani et al., 2016 [75]	General mental well-being	Cross-sectional	Iran	Mean age = 24.14	*N* = 541, Females: *n* = 403, Males: *n *= 138
Piqueras et al., 2011 [76]	General mental well-being	Cross-sectional	Chile	Mean age = 19.89 years	*N* = 3461, Females: *n* = 1595, Males: *n* = 1866
Schnettler et al., 2015 [77]	General mental well-being	Cross-sectional	Chile	Mean age = 20.9 years	*N* = 369, Females: *n* = 198, Males: *n* = 171
Chacon-Cuberos et al. 2019 [78]	Academic stress	Cross-sectional	Spain	Mean age = 21.58 years	*N* = 515, Females: *n* = 253, Males: *n* = 262
Chacon-Cuberos et al., 2018 [79]	Self-concept	Cross-sectional	Spain	Mean age = 22.2 years	*N* = 775, Females: *n* = 320, Males: *n* = 455
Zurita-Ortega et al., 2018 [80]	Self-concept	Cross-sectional	Spain	Mean age = 18.99 years	*N* = 597, Females: *n* = 44, Males: *n* = 156
Lutz et al., 2017 [81]	Psychological resilience	Cross-sectional	United States	Mean age = 21 years	*N* = 656, Females: *n* = 273, Males: *n* = 383

In order to describe the results, we organized the studies into those that used a diet quality tool as a predictor of diet quality and those that used other kinds of tools, such as food frequency questionnaires. We also organized the results in terms of outcomes, including depression, anxiety and stress (Table II).

**Table II.** Results (for studies investigating association of diet quality with mental health parameters)

								Hypothesis outcome			Effect size^a^		
Author, year	Diet quality tool	Depression tool	Dietary assessment	Model	Adjustment	Result	OR, HR or RR, β coefficients, or other statistics	1	2	3	Small	Medium	Large
Mental health parameter: depression													
Açik and Cakiroglu, 2019 [44]	DII	ZSRDS	3-day food records	Multivariate logistic regression analysis	Age, smoking, alcohol, PA level, anthropometric measurements	Poor diet quality was positively associated with depression scores	OR = 2.90 (95% CI 1.51–5.98)	X			X		
Jeffers et al., 2019 [45]	General estimating equations of dietary quality	PANAS	EMA	Generalized estimating equations	Each food item was examined as a predictor in separate models and each of the negative and positive effect was used as separate dependent variables	There was a positive association between fruits and positive affect (i). There was a positive association between sugary foods and negative affect (ii)	(i) Estimate = 1.37 (SE 0.49, *P* < 0.005) (ii) Estimate = 0.06 (SE 0.03, *P* < 0.02)	X			X		
Faghih et al., 2020 [46]	DASH	DASS-21	Semi-quantitative FFQ	Pearson’s correlation coefficients	Socio-economic, lifestyle and anthropometric characteristics	There was a negative correlation between diet quality and depression	Pearson’s coefficient = −0.434 (*P* < 0.001)	X				X	
Ramón- Arbués et al., 2019 [64]	HEI	DASS-21	N/A	Pearson’s correlation coefficients	Age, sex, study area, habitual residence, relationship status, height, weight, perceived economic situation, smoking, alcohol consumption, PA and sedentary lifestyle	There was no significant association between HEI and depression	N/A			X			
Attlee et al., 2022 [65]	E-DII	DASS-21	24-h dietary recall	Logistic regression analysis	Body habitus measures (BMI and WC), nutrient intakes and specific food groups, smoking status, PA categories	No significant association	N/A			X			
Lee et al., 2022 [63]	N/A	DASS-21	FFQ	Linear regression	Age, gender, ethnicity, relationship status, employment, income, living arrangements, number of children, education	The likelihood of more severe depression increased with higher consumption of grain (cereal) food (i) and lower consumption of dairy products (ii)	(i) β = 1.61, 95% CI, 0.22, 3.01 (ii) β = −3.38, 95% CI, −5.39, −1.38	X					X
Stanton et al., 2021 [62]	N/A	DASS-21	Previously validated Australian FFQ	Multivariate regression analysis	Gender, age, enrolment, ethnicity, relationship status, living arrangement, work, health conditions	Intake of snack foods was associated with higher depression scores	β = 8.66, *P* < 0.05	X					X
Abramson 2017 [119]	HEI	BDI	FFQ (5 days)	Spearman and partial correlations	Age, gender	There was no significant association between HEI and depression	N/A			X			
Quehl et al., 2017 [48]	HEI	CES-D	3-day food records	Linear regression	Age	Diet quality was negatively associated with depression scores	β= −0.016 (95% CI −0.029 to −0.003, *P *= 0.017)	X			X		
Sakai et al. 2017 [47]	DQS	CES-D	Diet history questionnaire	Multivariate analysis	BMI, current smoking, medication use, self-reported level of stress, dietary reporting status, PA, energy intake and living alone	Diet quality was negatively associated with depression	OR for depression in highest versus lowest quintiles of diet quality was 0.65 (95 % CI 0.50–0.84, *P* = 0.0005)	X			X		
Hamazaki et al., 2015 [50]	N/A	CES-D	Customary intake frequency	Multivariate logistic analysis	Age, gender, academic performance, friendships, financial matters, smoking status, consumption of alcohol, PA	Fish intake was negatively associated with depression	OR= 0.65, (95% CI 0.46–0.92) of highest versus lowest category of fish consumption	X			X		
Liu et al., 2007 [51]	N/A	CES-D	FFQ	Stepwise logistic regression	Gender, grade, city, perceived weight, smoking level and alcohol use	Risk of depression was increased with low fruit frequency and decreased with low ready to eat food, low snack food frequency and low fast food frequency.	OR for depression was 1.62 (*P* < 0.0001) for low fruit frequency, frequency, 0.70	X			X		
						BMI was not significantly associated with depression scores	(*P* < 0.0001) for low ready to eat food frequency, 0.73 (*P* < 0.05) for low snack food and 0.40 (*P* < 0.05) for low fast food frequency						
Peltzer and Pengpid, 2017a [52]	N/A	CES-D	FFQ	ANCOVA, descriptive statistics	Age, sex, subjective socio-economic status, country, BMI and PA	Fruit consumption was negatively associated with depression. Unhealthy dietary behaviours were positively associated with depression	Depression score was 13.28 for no fast food versus 13.70 for highest fast food consumption	X					
Peltzer and Pengpid, 2017b [53]	N/A	CES-D	FFQ	Stepwise multiple linear regression	Fruit and vegetable consumption, socio-demographic and health-related factors	Depression decreased with any increase in fruit and vegetable consumption	Strongest decrease in depression was with six servings of fruit and vegetables, *b* = −1.04 (*P* < 0.001)	X			X		
Smith-Marek et al., 2016 [54]	N/A	CES-D	Three items taken from the Family Transitions Project survey	Path analysis	Trauma, diet and exercise	A healthier diet was positively associated with lower depression scores	*b* = 2.57 (*P* < 0.001)	X			X		
Breiholz, 2010 [120]	N/A	CES-D	FFQ	Independent samples *t*-test	Gender	There was no association between high consumption of fruits/vegetables and depression	N/A						
El Ansari et al 2014 [55]	N/A	BDI	FFQ (12 items)	Regression analyses	University, sex	Unhealthy food was positively correlated with depression scores (i) Fruit/vegetable intake was negatively correlated with depression scores (ii)	(i) Coefficient = 0.072 for female, 0.158 for male. (ii) Coefficient = −0.081 for female, −0.115 for male	X			X		
Mikolajczyk et al., 2009 [59]	N/A	BDI	FFQ	Multivariable linear regression analysis	Gender and country	In females only, poor diet quality was positively associated with depression	Estimates for change in BDI per unit of food group frequency scale was −1.69 (*P* = 0.002), −1.62, (*P* = 0.003), −1.47 (*P* = 0.003) for less frequent consumption of fruits, vegetables and meat respectively	X			X		
Oleszko et al., 2019 [56]	N/A	BDI	FFQ (for 30 days before study)	Non-parametric Tau Kendall’s test	N/A	Diet quality was negatively associated with depression	Tau Kendall’s = −0.09 (*P* < 0.01)	X			X		
Rossa-Rocor et al., 2021 [49]	DSQ	PHQ-9	One item dietary preference	Multivariate regression analysis	Age, gender, ethnicity, PA, sleep, weight satisfaction, stress, stressful life events, social support	The junk food component was positively associated with depression	β = 0.26, *P* < 0.001	X				X	
Romijn, 2020 [57]	N/A	PHQ-9	FFQ	Pearson’s correlation coefficients	Gender, ethnicity, year of study, eating disorder	Diet quality was negatively associated with depression	Pearson’s coefficient = −0.38 (*P* < 0.001)	X				X	
Rossa- Roccor, 2019 [60]	N/A	PHQ-9	Posteriori self-reported diet	Multiple linear regression	Social support, PA, stress, body image and stressful life events	The processed food diet pattern was positively associated with depression scores (z-score β = 0.21, *P* ≤ .001).	z-score β= 0.21 (*P* ≤ 0.001)	X			X		
Jaalouk et al., 2019 [121]	N/A	PHQ-9	73-item FFQ	Multivariable linear regression analyses	Age, sex, income, PA, BMI, family history of mental illness, alcohol consumption, stressful life events, worrying about loss of control over how much they eat, use of antidepressants	There was no association of identified dietary patterns (traditional Lebanese, Western fast food, dairy, Lebanese fast food, fruits) with depression scores	N/A			X			
Tran et al., 2017 [61]	N/A	Clinical screening	Dietary questionnaire	Multivariate logistic regression models	Age, gender, blood pressure, heart rate, BMI, presence of depressive disorder, anxiety disorder and panic attack disorder	Poor diet quality was associated with increased risk for depression	OR 1.49 (*P* < 0.0001)	X			X		
Wattick et al., 2018 [58]	N/A	Centre for Disease Control and Prevention’s Healthy Days Measure	Dietary questionnaire	Logistic regression	Gender, housing and food security	Fruit and vegetable intake were negatively associated with depression in males	OR 0.68 (95% CI 0.50–0.89)	X			X		
Mental health parameter: anxiety													
Author, year	Diet quality tool	Anxiety tool	Dietary assessment	Model	Adjustment	Result	OR, HR or RR, β coefficients, or other statistics	Hypothesis outcome			Effect size^a^		
								1	2	3	Small	Medium	Large
Faghih et al., 2020 [46]	DASH	DASS-21	Semi-quantitative FFQ	Pearson’s correlation coefficients	Socio-economic, lifestyle, anthropometric characteristics	Diet quality was negatively associated with anxiety scores	Pearson’s correlation coefficient = −0.325 (*P* < 0.001)	X				X	
Ramón- Arbués et al., 2019 [64]	HEI	DASS-21	N/A	Pearson’s correlation coefficients	Age, sex, study area, habitual residence, relationship status, height, weight, perceived economic situation, smoking, alcohol consumption, PA and sedentary lifestyle	Diet quality was negatively associated with anxiety scores	Pearson’s correlation coefficient = −0.10 (*P* < 0.01)	X			X		
Attlee et al., 2022 [65]	E-DII	DASS-21	24-h dietary recall	Logistic regression analysis	Body habitus measures (BMI and WC), nutrient intakes and specific food groups, smoking status, PA categories	Each point increase in the E-DII score was associated with symptoms of anxiety	OR = 1.35; 95% CI: 1.07–1.69; *P* = 0.01	X			X		
Lee et al., 2022 [63]	N/A	DASS-21	FFQ	Linear regression	Age, gender, ethnicity, relationship status, employment, income, living arrangements, number of children, education	The likelihood of more severe anxiety increased with higher consumption junk food	β = 0.62, 95% CI: 0.01, 1.22	X					X
Rossa-Rocor et al., 2021 [49]	DSQ	GAD-7	One item dietary preference	Multivariate regression analysis	Age, gender, ethnicity, PA, sleep, weight satisfaction, stress, stressful life events, social support	The junk food component was positively associated with anxiety	β = 0.18, *P* = 0.001	X				X	
Romijn, 2020 [57]	N/A	GAD-7	FFQ	Pearson’s correlation coefficients	Gender, ethnicity, year of study, eating disorder	Diet quality was negatively correlated with anxiety scores	Pearson’s correlation coefficient = −0.31 *(P* < 0.001)	X				X	
Rossa-Roccor, 2019 [60]	N/A	GAD-7	Posteriori self-reported dietary patterns	Multiple linear regression	Social support, PA, stress, body image and stressful life events	The processed food diet pattern was positively associated with anxiety	β = 0.14 (*P* ≤ 0.001)	X			X		
Wattick et al., 2018 [58]	N/A	Centre for Disease Control and Prevention Healthy Days Measure	DSQ	Logistic regression	Gender, housing and food security	Higher added sugars intake was positively associated with anxiety in females	OR = 1.18 (95% CI 1.05–1.32)	X			X		
Tran et al., 2017 [61]	N/A	Clinical screening	Questionnaire about dietary behaviour	Multi variate logistic regression models	Age, gender, blood pressure, heart rate, BMI, presence/absence of depressive disorder, anxiety disorder and panic attack disorder	There was no association between bad dietary behaviour and anxiety.	N/A			X			
Mental health parameter: stress													
Author, year	Diet quality tool	Stress tool	Dietary assessment	Model	Adjustment	Result	OR, HR or RR, β coefficients, or other statistics	Hypothesis outcome			Effect size^a^		
								1	2	3	Small	Medium	Large
Faghih et al., 2020 [46]	DASH	DASS-21	Semi-quantitative FFQ	Pearson’s correlation coefficients	Socio-economic, lifestyle, anthropometric characteristics	Diet quality was negatively correlated with stress score	Pearson’s coefficient = −0.408 (*P* < 0.001)	X				X	
Saharkhiz et al., 2021 [68]	DASH score	DASS-21	FFQ	Multinomial logistic regression	Age, BMI, energy intake	Adherence to DASH style-pattern was associated with a lower stress score	OR = 0.32; 95% CI: 0.14–0.71, *P* = 0.009; second tertile with first DASH tertile	X				X	
Ramón- Arbués et al., 2019 [64]	HEI	DASS-21	N/A	Pearson’s correlation coefficients	Age, sex, study area, habitual residence, relationship status, height, weight, perceived economic situation, smoking, alcohol consumption, PA and sedentary lifestyle	Diet quality was negatively correlated with stress score	Pearson’s coefficient = −0.07 (*P* < 0.05)	X			X		
Attlee et al., 2022 [65]	E-DII	DASS-21	24 h dietary recall	Logistic regression analysis	Body habitus measures (BMI and WC), nutrient intakes and specific food groups, smoking status, PA categories	Each point increase in the E-DII score was associated with symptoms of stress.	OR = 1.41; 95% CI: 1.12–1.77; p = 0.003	X			X		
Stanton et al., 2021 [62]	N/A	DASS-21	Previously validated Australian FFQ	Multivariate regression analysis	Gender, age, enrolment, ethnicity, relationship status, living arrangement, work, health conditions	Intake of snack foods was associated with higher stress scores	β = 3.92, *P* = 0.055	X					X
Lee et al., 2022 [63]	N/A	DASS-21	FFQ	Linear regression	Age, gender, ethnicity, relationship status, employment, income, living arrangements, number of children, education	The likelihood of more severe stress increased with lower consumption of dairy products	β = −1.94, 95% CI, −3.65, −1.23	X					X
Fabian et al., 2013 [122]	Dietary guideline adherence index	27-item stress questionnaire	FFQ	Pearson’s chi-squared test	Age, gender, household income, school, BMI	Dietary patterns were not associated with stress levels	N/A			X			
Alfreeh et al., 2020 [67]	E-DII	PSS-10	FFQ (Saudi)	Multiple linear regression analyses	Age, marital status, education level, course, income, financial status, sleep, PA, previous weight reduction diet	Pro-inflammatory diets were associated with increased stress.	A higher E-DII score per 1 SD (1.8) was associated with 2.4-times higher PSS score. 95% CI: 1.8, 3.1 Pearson’s partial correlation coefficient of the relationship between E-DII scores and PSS scores was (*r*) 0.46	X				X	
El Ansari et al., 2015a [66]	Dietary guideline adherence index	PSS	12-item FFQ	Spearman rank coefficients	Age, sex, living situation, economic situation, moderate PA and BMI	Diet quality was negatively correlated to stress	Males: *r* = −0.21, *P* < 0.001 Females: *r* = −0.13, *P* < 0.001 Normal weight: *r* = −0.13, *P* < 0.001 Overweight: *r* = −0.21, *P* = 0.002	X			X		
El Ansari et al., 2014 [55]	N/A	PSS	12-item FFQ	Regression analyses	University, sex	Unhealthy foods were positively correlated with stress for females (i). Fruits and vegetables were negatively correlated with stress (ii)	(i) Coefficient = 0.051 (ii) Coefficient = −0.067 for female, −0.092 for male	X			X		
Liu et al., 2007 [51]	N/A	PSS	FFQ	Stepwise logistic regression	Gender, grade, city, perceived weight, smoking level and alcohol use	Low fruit frequency was positively correlated with stress (i). Low ready to eat food frequency (ii) and low snack food frequency (iii) were negatively correlated with stress. There was no association between BMI and stress scores	(i). OR = 1.53 (*P* < 0.01) (ii) OR = 0.69 (*P* < 0.01) (iii) OR = 0.75 (*P* < 0.05)	X			X		
Mikolajczyk et al., 2009 [59]	N/A	PSS	12-item FFQ	Multivariable linear regression analysis	Gender and country	In females only, consumption of sweets was positively associated with stress (i). In females only, consumption of fruits (ii) and vegetables (iii) was negatively associated with stress	(i) Estimate = 0.54 (*P* = 0.04) (ii) Estimate = −1.17 (*P* < 0.001) (iii) Estimate = −0.82 (*P* = 0.003)	X			X		
Lockhart, 2017 [123]	N/A	5-item emotional distress scale	FFQ	Multiple linear regression	Exercise and rest	No correlation between consumption of fruits and vegetables and emotional distress	N/A			X			
Mental health parameter: general mental well-being													
Author, year	Diet quality tool	Mental well-being tool	Dietary assessment	Model	Adjustment	Result	OR, HR or RR, β coefficients or other statistics	Hypothesis outcome			Effect size^a^		
								1	2	3	Small	Medium	Large
Aceijas et al., 2017 [39]	REAP-S	SWEMWBS	N/A	Multivariate analysis	Gender, lack of help-seeking behaviour in case of distress, negative attitudes towards nutrition-related activities, financial difficulties	Low diet quality almost doubled the risk of low mental well-being	OR = 1.7 (95% CI 1.0-2.7, *P* = 0 0.04).	X			X		
Lo Moro et al., 2021 [71]	MEDAS	WEMWBS	N/A	Linear regression analysis	Age, gender	The mental well-being and adherence to MD were positively associated	AdjB 0.676, 95% CI 0.277–1.075, *P* = 0.001	X					X
El Ansari et al., 2015b [69]	Dietary guideline adherence index	Assessment of self-reported health complaints (22 items)	12-item FFQ	Multi nomial logistic regression model	Age group, living situation, economic situation, PA, BMI	There was a negative correlation between diet quality and psychological health complaints	Beta coefficient = 0.06	X			X		
Hendy, 2012 [70]	Scores for total calories, carbohydrate percentage of calories, grams saturated fat and milligrams of sodium	PANAS	Anonymous 7-day record of foods	Multiple regression analyses	Restrained eating scores and gender	Consumption of calories (i), saturated fat (ii) and sodium (iii) was significantly associated with increased negative affect. There was no association for carbohydrate consumption	(i) *b* = 0.45 (ii) *b* = 0.43 (iii) *b* = 0.45	X			X		
Lopez- Olivares, 2020 [72]	PRE-DIMED Questionnaire	PANAS	N/A	Multiple regression models	Age, sex, PA, general state of health	A strict adherence to the MD was positively associated with positive emotional state. There was no association with negative emotional state	Coefficient = 0.018 (*P* = 0.009)	X			X		
Faghih et al., 2020 [46]	DASH	GHQ-12	Validated 168-item semi-quantitative FFQ	Pearson’s correlation coefficient	Socio-economic, lifestyle, anthropometric characteristics	Diet quality was positively correlated with mental health well-being	Pearson’s correlation coefficient = −0.431, (*P* < 0.001)	X				X	
Mochi- masu et al., 2016 [73]	N/A	GHQ-12	FFQ	Multiple regression analysis	BMI, PAL, energy and sucrose	Confectionaries intake was negatively associated with mental well-being and was the determining factor for the GHQ12 scores	*b* = 0.160, (*P* = 0.042)	X			X		
Knowlden et al., 2016 [74]	N/A	K-6	FFQ (24 h)	Pearson’s correlation and Cronbach alphas	Optimism, self-esteem and social support	Frequent fruit consumption (i) and infrequent consumption of sugar-sweetened beverages (ii) was associated with low levels of mental distress. No associations with BMI	(i) H2 = 7.268 (*P* = 0.026) (ii) H2 = 18.15 (*P* < 0.001)	X			X		
Lesani et al., 2016 [75]	N/A	Oxford Happiness Questionnaire	FFQ	ANCOVA	BMI, marital status, socio-economic status, PA, experience of stress in the last 6 months and having a defined disease	Amount of fruit and vegetable consumption was positively associated with mental well-being	*P* < 0.045 for 1 versus 3 servings per day	X					
Peltzer and Pengpid, 2017a [52]	N/A	SHS	FFQ	ANCOVA	Age, sex, subjective socio-economic status, country, BMI and PA	Diet quality was positively associated with happiness and high life satisfaction	SHS score was 2.87 for no fruit consumption versus 3.03 for consuming three fruits per day	X					
Piqueras et al., 2011 [76]	N/A	SHS	FFQ	Multi variate binary logistic regression	Gender, age, perceived stress and health behaviours	Intake of fruits and vegetables intake was positively associated with happiness	Adjusted OR = 1.34 (*P* = 0.000)	X			X		
Schnettler et al., 2015 [77]	N/A	SWLS	SWFL and FFQ	Dunnett’s T3 multiple comparisons test	Sex, age, residence, socio-economic factors	Students with healthful eating habits had higher levels of life satisfaction and satisfaction with food-related life	The group ‘satisfied with their life and their food-related life’ had a higher percentage of fruit (41.7%) and vegetable (57.6%) consumption daily	X					
Rossa- Rocor 2019 [60]	N/A	QOL Single item	Posteriori self-reported dietary patterns	Multiple linear regression	Social support, PA, stress, body image and stressful life events	There was no association between diet preference categories and mental well-being	N/A			X			
Mental health parameter: academic stress													
Author, year	Dietary score	Academic stress tool	Dietary assessment	Model	Adjustment	Result	OR, HR or RR, β coefficients or other statistics	Hypothesis outcome			Effect size^a^		
								1	2	3	Small	Medium	Large
Chacon-Cuberos et al. 2019 [78]	KIDMED	Validated Scale of Academic Stress	N/A	Regression model	Sex, BMI	MD adherence decreased stress in ‘Communication of own idea’ for high versus low MD adherence	F = 2.801 (*P* = 0.045)	X					X
Mental health parameter: self-concept													
Author, year	Dietary score	Self-concept tool	Dietary assessment	Model	Adjustment	Result	OR, HR or RR, β coefficients or other statistics	Hypothesis outcome			Effect size^a^		
								1	2	3	Small	Medium	Large
Chacon-Cuberos et al., 2018 [79]	KIDMED	AF-5	N/A	Structural Equation Model, Pearson Chi-square test)	Task and Ego Climate, Tobacco consumption, adherence to MD, PA, alcohol consumption, VO2MAX, Self-Concept, gender	MD was positively associated with self-concept	*b* = 0.08, (*P* < 0.05 for male) *b* = 0.17, (*P* < 0.01) for female)	X			X		
Zurita- Ortega et al., 2018 [80]	KIDMED	AF-5	N/A	Chi-square analysis and ANOVA	MD, PA, gender, religious belief, university campus and place of residence	Adherence to MD was positively associated with academic self-concept and physical self-concept. There were no associations for social, emotional and family self-concept	Academic self-concept (*P* = 0.001) and physical self-concept (*P* = 0.005) were more positive with high MD adherence (M = 3.67 and M = 3.39 respectively) compared with medium adherence (M = 3.45 and M = 3.16 respectively)	X					
Mental health parameter: psychological resilience													
Author, year	Dietary score	Psychological resilience tool	Dietary assessment	Model	Adjustment	Result	OR, HR or RR, β coefficients or other statistics	Hypothesis outcome			Effect size^a^		
								1	2	3	Small	Medium	Large
Lutz et al., 2017 [81]	HEI	CDRS	The Block FFQ	Logistic regression	Race, ethnicity, education, smoking, age, BMI, sex and military branch	Higher diet quality was associated with an increased likelihood of a participant being in the high-resilience group	OR 1.02 (95% CI 1.01–1.04)	X			X		
Mental health parameter: PTSD													
Author, year	Dietary score	PTSD tool	Dietary assessment	Model	Adjustment	Result	OR, HR or RR, β coefficients or other statistics	Hypothesis outcome			Effect size^a^		
								1	2	3	Small	Medium	Large
Peltzer and Pengpid, 2017a [52]	N/A	B7ISQ	Food frequency questionnaire (FFQ)	ANCOVA	Age, sex, subjective socio-economic status, country, BMI and PA	Fruit consumption were negatively associated with traumatic stress symptoms	B7ISQ scores were 19.25 for consumption of 4 or more fruits versus 19.91 for no fruit consumption	X					
Smith- Marek et al., 2016 [54]	N/A	PCL-5	Three items taken from the Family Transitions Project survey	Path analysis	Trauma, diet and exercise	A healthier diet was significantly associated with lower post-traumatic stress scores	b = 1.60 (*P* < 0.01)	X			X		

Note: Studies ordered according to diet quality tool used; if no diet quality tool used, studies were ordered according to depression tool.

Dietary measures: Diet inflammatory score (DII), Dietary Approaches to Stop Hypertension score (DASH), Energy-adjusted Dietary Inflammatory Index (E-DII), Healthy eating index (HEI), Diet quality score (DQS), Food frequency questionnaire (FFQ), Ecological Momentary Assessment (EMA), Dietary Screener Questionnaire (DSQ), Rapid Eating and Activity Assessment for Patients-Short Version (REAP-S), PREvención con DIeta MEDiterránea questionnaire (PREDIMED), Satisfaction with Food-related Life Scale (SWFL), Test of Adherence to Mediterranean Diet (KIDMED), Mediterranean diet (MD), Physical Activity (PA), Body Mass Index (BMI).

Mental health scores: Zung Self-Rating Depression Scale (ZSRDS), Positive and Negative Affect Scale (PANAS), Depression, anxiety and stress scale (DASS-21), Beck Depression Inventory (BDI), Centre for Epidemiologic Studies Depression Scale (CES-D), Patient health questionnaire (PHQ-9), Cohen’s Perceived Stress Scale (PSS), General anxiety disorder 7 (GAD-7), Warwick–Edinburgh Mental Wellbeing Scale short version (SWEMWBS), Positive and Negative Affect Scale (PANAS), 12-item general health questionnaire (GHQ-12), Kessler-6 Psychological Distress Scale (K-6), Subjective happiness scale (SHS), Satisfaction with Life Scale (SWLS), Connor-Davidson Resilience Scale (CDRS), Breslau’s 7-item screening questionnaire (B7ISQ), Post-traumatic stress Checklist (PCL-5), Five-Factor Self-Concept Questionnaire (AF-5).

Statistics: Odds Ratio (OR), Hazard Ratio (HR), Relative Risk (RR), Confidence Interval (CI), Standard Error (SE), Analysis of covariance (ANCOVA), Between group differences (H2), Mean (M), Regression coefficient (F).

Not applicable (N/A).

Hypothesis: Good diet quality will have a beneficial effect on mental health parameters, and/or bad diet quality will have a detrimental effect on mental health parameters.

Hypothesis outcomes:

(i) Hypothesis accepted.

(ii) Hypothesis rejected—good diet quality had an adverse effect on mental health.

(iii) Hypothesis rejected—no association between diet quality and mental health.

a If applicable.

There were 25 studies that investigated the influence of diet quality on depression. Of these studies, 20 suggested diet quality to be negatively associated with depression. Out of the nine studies that used a diet quality score, six found a significant negative association of diet quality with depression scores [44–49]. The remaining studies used questionnaires, and there was evidence to suggest that healthy diet was associated with lower depression scores [50–58], as well as that unhealthy diet was associated with higher depression scores [51, 52, 55, 57, 59–63].

Nine studies examined the influence of diet quality on anxiety, of which eight found significant associations. Of these studies, four used a diet quality measure and all found a negative association of diet quality with anxiety [46, 49, 64, 65]. Studies using questionnaires also showed that unhealthy diet was positively associated with anxiety [57, 58, 60, 63].

Thirteen studies looked into the influence of diet quality on stress, of which 11 found significant associations. Six studies using a diet quality measure found a negative association of diet quality with stress [46, 64–68]. Additionally, studies using questionnaires found that unhealthy diets were positively correlated with stress [51, 55, 59, 62, 63], as well as that healthy diets were negatively correlated with stress [51, 55, 59].

There were 13 identified studies investigating the influence of diet quality on general mental well-being, of which 12 found significant associations. Out of the six studies that used a diet quality measure, four concluded that poor diet quality was negatively associated with mental well-being [39, 46, 69, 70], and one concluded that good diet quality was positively associated with mental well-being [71]. One study found a positive association of diet quality with positive emotional state, but no association with negative emotional state [72]. Studies using questionnaires reported unhealthy diets to be associated with bad mental well-being [52, 73, 74], as well as healthy diets to be associated with good mental well-being [52, 74–77].

Other mental health parameters that were examined by studies to determine whether they are influenced by diet included post-traumatic stress disorder, academic stress, positive self-concept and psychological resilience. All of these studies reported results towards the expected direction. Specifically, two studies reported that a healthier diet was associated with fewer post-traumatic stress symptoms in university students [52, 54], one study reported that high adherence to the Mediterranean diet decreased academic stress in regard to students communicating their own ideas [78], two studies reported that the Mediterranean diet was associated with more positive self-concept [79, 80] and one study suggested that better diet quality was associated with better psychological resilience [81].

In terms of effect sizes for studies investigating the association of diet quality with mental health, it was possible to retrieve information for 31 out of the 35 studies that found significant associations (Table II). It was observed that effect sizes were small for 22 studies, moderate for five studies and large for four studies (Table II).

### Mental health associations with diet quality

Twenty-two primary studies and one systematic literature review investigated the associations of mental health with diet quality. The identified studies took place in various locations (Table III). Specifically, 32% of the studies were conducted in the United States and Canada, 29% in the Middle East, 14% in Europe, 10% in Latin America, 10% in Asia and 5% in Australia. The number of study participants varied from 88 to 2810 (excluding the systematic literature review). The main findings of these studies are summarized in Table IV.

**Table III.** Baseline characteristics (for studies investigating association of mental health parameters with diet quality)

Author, year	Mental health parameter	Country	Design	Age details	N students, sex
Hall et al., 2017 [84]	Depression, anxiety	Mexico	Cross-sectional	Mean age = 21 years	*N* = 450, three-quarters were females
Lazarevich et al., 2018 [82]	Depression	Mexico	Cross-sectional	Mean age = 19.6 years	*N* = 1104, Females: *n* = 659, Males: *n* = 445
Dalton & Hammen, 2018 [91]	Depression, stress	United States	Longitudinal	Mean age = 19.11 years	*N* = 127, Females: *n* = 100, Males: *n* = 26, Other: *n* = 1
Keck et al., 2020 [83]	Depression, stress	United States	Cross-sectional	Mean age = 18.91 years, Age range = 18–25 years	*N* = 225, Females: *n* = 139, Males: *n *= 86
Kotecki et al., 2019 [86]	Stress	United States	Cross-sectional	Age range = 18-20 years	N = 1198, Females: *n* = 791, Males: *n* = 407
El Ansari and Berg-Beckhoff, 2015 [85]	Stress	Egypt	Cross-sectional	Age range = 16–30 years	*N* = 2810, Females: *n* = 1483, Males: *n* = 1327
Leblanc and Villalon, 2008 [124]	Stress	Canada	Longitudinal	Age range = 19–22 years	*N* = 94 at start, *N* = 63 at end, Females: 83%, Males: 17%
Natascin and Fiocco, 2015 [95]	Stress	Canada	Cross-sectional	Age details not available	*N* = 136, Females: *n* = 111, Males: *n* = 19
Peker and Bermek, 2011 [87]	Stress	Turkey	Cross-sectional	Mean age = 19.43 years	*N* = 111, Females: *n* = 56, Males: *n* = 55
Cheng and Mohd Kamil, 2020 [125]	Stress	Malaysia	Cross-sectional	Mean age = 21.27 years	*N* = 100, Females: *n* = 50, Males: *n* = 50
Ahmed et al., 2014 [88]	Stress	Kuwait	Cross-sectional	Aged ≥ 18 years	*N* = 407, Females: *n* = 164, Males: *n* = 243
Almogbel et al., 2019 [89]	Stress	Saudi Arabia	Cross-sectional	59.8% between 18 and 20 years	*N* = 614, Females: *n* = 220, Males: *n* = 394
Papier et al., 2015 [90]	Stress	Australia	Cross-sectional	Mean age = 21.5 years	*N* = 728, Females: *n* = 397, Males: *n* = 331
Errisuriz et al., 2016 [92]	Stress	United States	Cross-sectional	Mean age = 18.9 years	*N* = 736, Females: *n *= 433, Males: *n* = 303
Kandiah et al., 2006 [93]	Stress	United States	Cross-sectional	Age range 17–26 years	*N* = 272, All females
Oliver and Wardle, 1999 [94]	Stress	United Kingdom	Cross-sectional	Mean age = 24.4 years	*N* = 212, Females: *n* = 149, Males: *n* = 63
Carlos et al., 2020 [97]	Anxiety	Spain	Cross-sectional	Mean age 21.42 years, SD 4.73	*N* = 252, Females: *n* = 191, Males: *n* = 61
Pollard et al., 1995 [99]	Test anxiety	United Kingdom	Case–control study	Mean group age range = 21.7–23.8 years	*N* = 180, Females: *n* = 100, Males: *n* = 80
Trigueros et al., 2020 [98]	Test anxiety, academic stress	Spain	Cross-sectional	Mean age = 23.58 years	*N* = 1347, Females: *n *= 733, Males: *n* = 614
Aljaber et al., 2019 [101]	Academic stress	Saudi Arabia	Cross-sectional	Mean age = 21 years	*N* = 105, All males
Mansoury et al., 2015 [100]	Academic stress	Saudi Arabia	Longitudinal	Median age 21.6 years	*N* = 491 at start, *N* = 322 end, All females
Bu et al. 2019 [102]	Menstrual distress	China	Cross-sectional	Mean age = 21 years	*N* = 88, All females

**Table IV.** Results (for studies investigating association of mental health parameters with diet quality)

								Hypothesis outcome			Effect size^a^		
Author, year	Diet quality tool	Depression instrument	Dietary assessment	Model	Adjustment	Result	OR, HR or RR, β coefficients, or other statistics	1	2	3	Small	Medium	Large
Mental health parameter: depression													
Hall et al., 2017 [84]	Macronutrient and micronutrient scores (based on dietary guideline)	GDAAS	24-h dietary recalls	Binary logistic regression	University, diet perception, breakfast consumption, energy intake, soda consumption, weekly vigorous exercise	Depression had no effect on diet quality scores	N/A			X			
Keck et al., 2020 [83]	HEI	PHQ-9	ASA24	Multigroup path analysis	Race, marital status, college status, GAD-7 severity, PHQ-9 severity	There was a significant association of adverse PHQ-9 score with decreased total caloric intake and increased sugar intake. There was no effect on total HEI score	Total caloric intake: *b* = −27.44, SE 10.67, *P* < 0.01 Sugar HEI component: *b* = −0.17, SE = 0.05, *P* < 0.001	X				X	
Laza-revich et al., 2018 [82]	N/A	CES-D	69-item FFQ	Logistic regression analysis	Sex, age, BMI	In women, the fourth quartile of depression score was positively associated with frequent consumption of fast food (i), fried food (ii) and sugary food (iii). There were no associations for men	(i) OR = 2.08 (95% CI 1.14 −3.82, *P* = 0.018) (ii) OR = 1.92, (95% CI 1.17–3.15, *P* = 0.010) (iii) OR = 2.16, 95% CI 1.37–3.43, *P* < 0.001)	X			X		
Dalton and Hammen, 2018 [91]	N/A	BDI	Standard measures of daily eating habits	Hierarchical generalized linear modelling (Poisson)	Gender	There was no association of depression with daily maladaptive behaviours (including diet habits)	N/A			X			
Mental health parameter: anxiety													
								Hypothesis outcome			Effect size^a^		
Author, year	Diet quality tool	Anxiety tool	Dietary assessment	Model	Adjustment	Result	OR, HR or RR, β coefficients, or other statistics	1	2	3	Small	Medium	Large
Hall et al., 2017 [84]	Macronutrient and micronutrient scores, based on US dietary guidelines	Anxiety: Goldberg depression and anxiety scales	24-h dietary recall	Binary logistic regression	University, diet perception, breakfast consumption, energy intake, soda consumption and weekly vigorous exercise	Anxiety was associated with greater risk of low macronutrient quality	OR 2.35 (95% CI 1.27, 4.38)	X			X		
Carlos et al., 2020 [97]	KIDMED	STAI	N/A	Multiple regression analysis	Adhesion to the MD, alcohol consumption, level of emotional eating	Adhesion to the MD was not predicted by state anxiety	N/A			X			
Keck et al., 2020 [83]	HEI	GAD-7	ASA24	Multigroup path analysis	Race, marital status, college status, GAD-7 severity, PHQ-9 severity	There was a significant association of adverse GAD-7 score with decreased total caloric intake and increased sugar intake. There was no effect on total HEI score	Total caloric intake: b = −30.16, SE = 10.67, *P* < 0.01 Sugar HEI component: b = −0.16, SE = 0.05, *P* < 0.001	X				X	
Mental health parameter: stress													
								Hypothesis outcome			Effect size^a^		
Author, year	Diet quality tool	Stress tool	Dietary assessment	Model	Adjustment	Result	OR, HR or RR, β coefficients or other statistics	1	2	3	Small	Medium	Large
Kotecki et al., 2019 [86]	Online questionnaire assessing diet quality	Online questionnaire assessing perceived stress	N/A	Regression, ANOVA, ANCOVA	N/A	Perceived stress was negatively associated with diet quality	Mean diet quality scores were 51.32 for low perceived stress versus 50.17 for high perceived stress (*P* < 0.05)	X					
El Ansari and Berg- Beckhoff, 2015 [85]	Dietary Guideline Adherence Index	PSS	FFQ	Multiple linear regression models	Age, sex, living situation (accommodation during term time), economic situation, BMI, physical activity, faculty	Higher perceived stress score was significantly associated with less frequent food intake of fruit and vegetables. There was no significant association between unhealthy foods and stress	b = −0.12	X			X		
Daigle Leblanc and Villalon, 2008 [124]	N/A	PSS	Three-day food record and a FFQ	Pearson’s correlations, Student’s *t*-tests	N/A	Increased stress was associated with an increased consumption of milk and milk products for 1st-year students at the beginning of the first trimester (i) and of breads and cereals for 4th-year students at the end of the first trimester (ii)	(i) *P* = 0.05 (ii) *P* = 0.02	X					
Nataskin and Fiocco, 2015 [95]	N/A	PSS	Eating habits confidence scale and Block fat and sodium screener	Linear regression analyses	Perceived stress, diet self-efficacy, age, race and sex	Low levels of perceived stress were associated with the lowest levels of fat and sodium intake	b = −1.07 (*P* = 0.04)	X			X		
Peker and Bermek, 2011 [87]	N/A	PSS	Nutrition section of HPLP II	Pearson’s product moment correlation and stepwise multiple linear regression analysis	Age, place of residence, monthly family income, perceived social support and perceived stress	Perceived stress was negatively associated with healthy diet	*r* = −0.36 (*P* < 0.01)	X				X	
Cheng and Mohd Kamil, 2020 [125]	N/A	PSS	FFQ and 3-day dietary record	Independent samples *t*-tests and chi-square tests	N/A	There was no significant difference for all food categories between the non-stressed and stressed groups	N/A			X			
Ahmed et al., 2014 [88]	N/A	DASS-21	7-day FFQ	Logistic regression analysis	Age, year of study, family income, parents’ education level, marital status, smoking status	Stressed female students were more likely to eat fast foods (i) snacks and beverages (ii) than unstressed female students. No associations were found for males	(i) OR 1.75 (95% CI: 1.02–3:00) (ii) OR 2.28 (95% CI: 1.30–3.98)	X			X		
Almogbel et al. 2019 [89]	N/A	DASS-21	FFQ	Chi-square tests	N/A	Stressed participants consumed more junk foods. Non-stressed participants preferred healthier foods	*P* < 0.05	X					
Papier et al., 2015 [90]	N/A	DASS-21	CSIRO FFQ	Logistic regression analysis	Marital status, study status, living situation, working hours, frequency of exercise, BMI, whether participants were trying to lose weight, smoking status	Stress was negatively associated with consumption of meat alternatives, vegetables and fruits. Stress was positively associated with the consumption of highly processed food	OR 2–3, *P* < 0.05	X			X		
Dalton and Hammen, 2018 [91]	N/A	LSI and Daily Stress Measure	Standard measures of daily eating habits	Poisson linear modelling	Gender	Daily stress (i) and chronic stress (ii) were significantly associated with daily maladaptive behaviours (including unhealthy diet)	(i) *b* = 0.01 (*P* = 0.02) (ii) *b* = 0.02 (*P* = 0.03)	X			X		
Errisuriz et al., 2016 [92]	N/A	Stress: measured by single item Stress management: measured by single item	FFQ	Multiple hierarchical linear regressions	Gender, BMI and race	Perceived stress was positively associated with past week soda (i), coffee (ii), energy drink (iii), salty snack (IV), frozen food (v) and fast food consumption (vi)	(i) *b* = 0.09 (ii) *b* = 0.15 (iii) *b* = 0.14 (iv) *b* = 0.12 (v) *b* = 0.15 (vi) *b* = 0.09 (all *P* < 0.05)	X			X		
Kandiah et al., 2006 [93]	N/A	45-itemized stress-eating survey	45-itemized stress-eating survey	ANOVA	N/A	Only 33% ate healthy when stressed (compared to 80% when not stressed). When stressed, sweet foods were chosen	N/A	X					
Oliver and Wardle, 1999 [94]	N/A	Stress- induced eating Questionnaire	Stress- induced eating Questionnaire	Descriptive statistics, chi-squared test	Dieting status, gender	Intake of ‘snack-type’ foods increased during periods of stress; females were more likely to consume sweets and chocolate (I). Intake of ‘meal-type’ foods (fruit and vegetables, meat and fish) decreased during stressful periods	(i) Chi-squared = 10.9 (*P* < 0.01)	X					
Mental health parameter: test anxiety													
								Hypothesis outcome			Effect size^a^		
Author, year	Diet quality tool	Test anxiety tool	Dietary assessment	Model	Adjustment	Result	OR, HR or RR, β coefficients, or other statistics	1	2	3	Small	Medium	Large
Pollard et al., 1995	N/A	State anxiety scale	24-h dietary recall	Repeated measures analysis of covariance	Group (examination stress, control, gender, time, year of study, trait anxiety, social support, dietary restraint)	Students with high trait increased their consumption of total fat, saturated fat and total energy intake between baseline and examination sessions	*P* < 0.05	X					
Trigueros et al., 2020 [98]	KIDMED	The Test Anxiety Inventory	N/A	Structural equation model	Exam anxiety, academic stress, emotional intelligence, resilience	Exam anxiety negatively predicted adherence to the MD	Coefficient = 0.37 (*P* < 0.001)	X			X		
Mental health parameter: academic stress													
								Hypothesis outcome			Effect size^a^		
Author, year	Diet quality tool	Academic stress tool	Dietary assessment	Model	Adjustment	Result	OR, HR or RR, β coefficients, or other statistics	1	2	3	Small	Medium	Large
Trigueros et al., 2020 [98]	KIDMED	Student Stress Inventory Stress Manifestation	N/A	Structural equation model	Exam anxiety, academic stress, emotional intelligence, resilience	Academic stress negatively predicted adherence to the MD	Coefficient = 0.49, (*P* < 0.01)	X			X		
Aljaber et al., 2019 [101]	CES	ASS	N/A	Authors used 12 statements to test the hypothesis that students with high stress levels would eat more unhealthy foods	N/A	11 statements proved the hypothesis, only one statement disproved; the authors accepted the hypothesis that students who have a high stress level eat more unhealthy foods	N/A	X					
Mansoury et al. [2015, 100]	N/A	PSS	24-h recall food diary analysed using Diet Plan	*t*-tests	N/A	Participants experiencing academic stress at T2 demonstrated significantly lower frequency of healthy food intake at T2 compared to T1	*P* = 0.001	X					
Mental health parameter: menstrual distress													
Author, year	Diet quality tool	Menstrual distress tool	Dietary assessment	Model	Adjustment	Result	OR, HR or RR, β coefficients, or other statistics	Hypothesis outcome			Effect size^a^		
								1	2	3	Small	Medium	Large
Bu et al. 2019 [102]	N/A	MDQ	15-item FFQ	Multiple logistic regression analyses	N/A	Negative mood was positively associated with tea, coffee and carbonated beverage intake during the menstrual phase (I). Negative mood was positively associated with banana and dates intake during the premenstrual phase (ii)	(i) *b* = 0.21, *P* = 0.0453, OR = 1.23 (ii) *b* = 0.59, *P* = 0.0172, OR = 1.81	X			X		

Note: Studies ordered according to diet quality tool; if no diet quality tool used, studies were ordered according to depression instrument.

Dietary scores: Healthy Eating Index (HEI), Automated Self-Administered 24-h recall (ASA24), Food frequency questionnaire (FFQ), Mediterranean diet (MD), Health-promoting lifestyle II (HPLP II), Commonwealth Scientific and Industrial Research Organization food frequency questionnaire (CSIRO FFQ), Test of Adherence to Mediterranean Diet (KIDMED), Compulsive eating scale (CES).

Mental health scores: Goldberg depression and anxiety scales (GDAAS), Patient Health Questionnaire 9-Item (PHQ-9), Generalized Anxiety Disorder 7-items (GAD-7), Centre for Epidemiologic Studies scale (CES-D), Beck’s depression inventory (BDI), Depression, anxiety and stress scale (DASS-21), UCLA Life Stress Interview (LSI), Academic Stress Scale (ASS), Cohen’s perceived stress scale (PSS), Menstrual Distress Questionnaire (MDQ), State-Trait Anxiety Inventory (STAI).

Statistics: Odds Ratio (OR), Hazard Ratio (HR), Relative Risk (RR), M (mean), Analysis of variance/covariance (ANOVA/ANCOVA), Pearson’s coefficient (r), Beta coefficient (b), Standard Error (SE).

Not applicable (N/A).

Hypothesis: Good mental health will have a beneficial effect on diet quality, and/or bad mental health will have a detrimental effect on diet quality.

Hypothesis outcomes:

(i) Hypothesis accepted.

(ii) Hypothesis rejected—good mental health had an adverse effect on diet quality.

(iii) Hypothesis rejected—no association between mental health and diet quality.

a If applicable.

Out of the four studies investigating the influence of depression on diet quality, two found significant associations. In particular, one study found evidence that depressed women were more likely to follow unhealthy diets; however, no associations were found for men [82]. Moreover, one study found that increased sugar intake was associated with more symptoms of depression, but there were no associations of depression scores with the overall diet quality scores [83, 84].

Thirteen studies investigated the influence of stress on diet quality, of which 12 found significant associations. Of the studies that showed significant associations, two used a diet quality tool and found that high perceived stress was associated with low diet quality scores [85], as well as that low perceived stress was associated with high diet quality scores [86]. The remaining 10 studies used questionnaires and similarly showed that high perceived stress was associated with unhealthy diets [87–94], as well as that low perceived stress was associated with healthy diets [89, 95]. Moreover, evidence from two longitudinal studies suggested that increasing stress over a period of time can be detrimental on diet [91, 96].

Other mental health conditions that have been investigated to determine whether they can influence diet quality included anxiety, test anxiety, academic stress and menstrual distress. In particular, three cross-sectional studies investigated the influence of anxiety on diet quality by using a diet quality tool. One found that anxiety was associated with a greater risk of low macronutrient quality [84]; the other two studies found no associations of stress with the overall diet quality score [83, 97].

In terms of the influence of test anxiety and academic stress on diet quality, all four studies showed significant associations with diet quality [98–101]. Specifically, it was found that test anxiety and academic stress negatively predicted adherence to the Mediterranean diet [98]; there was also evidence from two longitudinal studies to suggest that as test anxiety/academic stress increased, the intake of unhealthy food also increased [99, 100]. The above findings were also supported by the results of a cross-sectional study [101].

Finally, one study investigated the influence of menstrual distress on diet [102] and found that negative mood during the menstrual/premenstrual phases was associated with diet changes. For example, negative mood was positively associated with ingestion of tea, coffee and carbonated drinks, as female students may have been trying to stimulate their nervous system to alleviate their negative mood through these diet changes.

In terms of effect sizes, it was possible to retrieve information about effect sizes for 10 studies (Table IV). The effect sizes observed were small for eight studies and moderate for two studies.

### Review papers

No previous systematic reviews appraising both directions of the association of diet quality with mental health were identified.

One previous review [33] aimed to examine the influence of diet on depression and anxiety among college students. In contrast to our review, this review filtered out articles published before 2000, and only included students enrolled in at least 2-year programmes. Moreover, this review focussed only on depression and anxiety, but no other aspects of mental health. This review assessed 16 cross-sectional studies, of which 14 fulfilled our inclusion criteria and have also been included in our review [44, 47, 48, 50, 53–55, 58, 59, 73, 82–84]. The results of this review were in line with our results, as the authors concluded that most of the cross-sectional studies found a positive influence of healthy diets on depression and anxiety, with a few studies finding inconsistent results.

One previous systematic review appraising the influence of stress on the diet of students [20] was identified. In contrast to our review, the authors reviewed the influence of stress but no other mental health disorders. The authors also included studies involving disordered eating and maladaptive weight-related behaviours, which was not the scope of our review. The stress and dietary intake section of the review identified 12 studies, all of which were considered to be of relevance and have been included in the current review [51, 55, 59, 66, 85, 87, 90, 92–95, 99]. The conclusions of this review were in line with our finding that there is a positive association of stress with unhealthy diet, as well as a negative association of stress with healthy diet in university students.

## Discussion

### Main findings

Sixty-six primary studies and two systematic literature review were reviewed in total. Majority of primary studies (*n* = 53) and the two reviews showed results in the expected direction, where good diet quality was associated with good mental health, and good mental health was related with good diet quality. In terms of dietary parameters, students consuming high-quality foods (including fruits, vegetables, nuts and fish) reported fewer mental health symptoms, compared with students who had a high intake of pro-inflammatory foods (such as processed meat, refined carbohydrates, desserts and sweetened beverages), who reported more mental health issues. 36 out of 45 studies supported this association. This was in line with the stress–diathesis model, with the unhealthy foods acting as the stressor and being associated with the development of mental health difficulties.

From a mechanistic point of view, there are various processes that could be mediating the relationship between diet quality and mental health. These include inflammation which is a core aetiological feature of depression [103]; various components of a healthy diet may be reducing inflammation [104]. Other candidate biological mechanisms include oxidative stress [105], maintenance of beneficial gut microbes [106], adult hippocampal neurogenesis [107], modulation of the tryptophan-kynurenine metabolism [108], maintenance of mitochondrial biogenesis [109] and regulation of epigenetic processes [110, 111].

No studies found an adverse influence of good diet quality nor a beneficial influence of bad diet quality on mental health. These results confirm that unhealthy diets such as western diets have no benefits on the mental health of students, which is in line with the findings of previous studies [107].

Majority of studies (19 out of 23) also showed associations of mental health with diet quality, which were in the expected direction. The most compelling evidence was in favour of high levels of stress having an unhealthy influence on diet. Despite a lack of longitudinal studies, the four identified longitudinal studies that either investigated the influence of stress or academic stress on diet quality showed that stress is associated with unhealthy diets over time. These results are in line with previous research in adults, which showed that stress was associated with unhealthy eating [112], including intake of foods with high sugar content.

A potential mechanism mediating this association may be the hypothalamus–pituitary–adrenal axis being hyperactive in depression and anxiety, leading to an increase in serum cortisol. As a consequence, appetite may be increased with a preference for energy-dense foods at the expense of healthy foods [113, 114]. Stimulation of the appetite-stimulating hormone ghrelin during stress may also be of relevance [115].

The most common country of study origin was the United States, where the characteristics of university life may be different than other developed or third world countries. However, studies in countries of lower socio-economic status were included in the review achieving good global coverage.

In terms of interpreting findings in the context of the stress–diathesis model, some of the studies recognized the complex relationships of biopsychosocial factors on both mental health and diet. Examples of such factors affecting mental health that we identified included stressful life events, body image, physical activity, sleep, social support, use of alcohol or illicit drugs. We also identified factors linked to diet, such as availability and access of pre-prepared meals/fast foods on campus, limited resources including money for shopping, no easy access to healthy food and lack of companionship during meal times.

The review identified that more studies used food frequency questionnaires rather than diet quality measures. This means when only food frequency questionnaires were used, it was not always possible to obtain a clear picture in regard to the extent that diets of participants conformed to dietary recommendations. In contrast, scores of validated diet quality instruments based on dietary recommendations were easier to interpret and contributed to a better understanding of the associations between diet quality and mental health.

### Limitations

Strength of this review is the fact that an exhaustive review of the literature was performed, including grey literature. However, it is not possible to exclude the possibility of having missed studies due to publication bias, in cases where non-significant results had not been published.

Some studies included participants who were 17 years old, which would classify as adolescents. Even though these studies were limited, the possibility of an age bias from these studies cannot be excluded.

This review largely relied on cross-sectional studies, which assessed diet and mental health at a single point of time. This means that it has not always been possible to make definite conclusions about the direction of associations or about the changes in diet quality or mental health over time.

### Implications for health education practice

Even though the effect sizes of the included studies were mostly small or moderate, the observed results still have implications in health education practice. Given the evidence that unhealthy dietary practices are associated with worsening mental health of students, mental health education at university should aim to raise awareness of this association. This could be done along with input from nutritionists and could involve the introduction of relevant modules in health courses, online courses, as well as the use of leaflets and posters in campuses [20].

This review has not identified any relevant whole-diet intervention or randomized controlled studies to improve mental health of students through diet, or vice versa. Results for an ongoing randomized controlled trial [116] are awaited, which involves the use of a web-based wellness platform to support healthy living of students by focussing on nutrition and physical activity.

An approach involving web interventions or lectures could also be followed to educate students about the relation of stress with unhealthy dietary habits. It has been suggested that the introduction of mindfulness-based stress reduction techniques and mindful eating may be effective techniques to address this issue [20, 117].

Universities may need to review how they operate, what economic and planning decisions they make in terms of which franchises they allow onto campuses, influence what is sold in shops and raise awareness of the importance of diet quality. By helping students improve their diet quality, they may experience fewer mental health issues, as well as fewer and less severe depressive episodes. Similarly, by helping students deal with stress, they may be able to experience healthier dietary habits during their university education.

### Future recommendations

Most of the identified studies were cross-sectional, as they assessed diet and mental health at a single point of time. We recommend that future studies use a longitudinal design when possible, enabling researchers to determine the direction of any detected associations. However, we recognize that longitudinal studies can pose challenges in terms of time and costs required.

Apart from the available cross-sectional studies establishing some associations, we also need well-powered clinical trials to further assess the associations of diet quality with the prevention, severity and relapse of depression, stress and other mental health issues of students. The findings would inform the design of further studies including randomized controlled trials and intervention studies. Such studies may provide more insight about the relationship between the three factors of stress, diet and emotion. However, it is recognized that designing and executing such studies may be challenging and that difficulties including randomization may be anticipated.

We used the stress–diathesis model to interrogate the data, where the diatheses could be of biological, psychological or social nature. We noticed that studies did not always include data relevant to this model. For example, biological factors such as genetic predispositions or gut microbiota of students were not reported. Psychological factors such as perfectionism traits were also not reported. Moreover, social factors were not always reported, such as lack of ability to form group memberships, lack of culinary and basic nutritional knowledge and lack of resources and access to healthy food. Hence, the included studies only partially covered the stress–diathesis model, meaning there is scope for future studies to use the stress–diathesis model as a reference.

In terms of dietary instruments, the HEI was the most common instrument used; however, it is a long instrument and students may not always engage. A previous study assessing the relationship of diet quality and mental health in adolescence has highlighted the need of a brief, validated measure of diet quality to be used in studies involving adolescents or young adults [118]. An example of such a measure might be the short-form food frequency questionnaire [17], which may maximize the student engagement for future studies. In terms of mental health measures, the DASS-21 was the most commonly used measure and is a good option as it provides information about the three mental health aspects of depression, anxiety and stress. Results of studies with consistent tools for diet quality and mental health might enable the execution of a meta-analysis in the future.

## Conclusions

The review results show observational evidence that a healthy diet of university students is associated with better mental health, as students who scored favourably in the diet quality instruments also scored favourably in depression, anxiety and stress scales. The opposite applies for university students following unhealthy diets, as unhealthy diet in this group of students is associated with depression, stress, anxiety and other mental health issues. There is also observational evidence to suggest that stress experienced by university students is associated with deterioration in their diet quality, including a reduction in the intake of fruits and vegetables and an increase in the consumption of sweets and fast food.

In order to establish the effectiveness of potential interventions for maintaining a healthy diet and good mental health of students, further observational studies, as well as randomized control trials, would be required. This would allow the determination of whether interventions to improve diet quality at the university level could reduce mental health issues and whether providing support to students under stress may lead to healthier dietary habits when living on campuses.

## Supplementary Material

cyac035_Supp
