# Supplementary material for: A systematic review of the association of diet quality with the mental health of university students: implications in health education practice
Source: Health Educ Res. 2022 Nov 28;38(1):28–68. doi: 10.1093/her/cyac035 (PMC9853940; doi:10.1093/her/cyac035)
Supplement: cyac035_Supp [file cyac035_supp.zip › (d) Online Supplementary materials.doc]

**Online supplementary material 1: Quality assessment (diet effects on mental health), based on Newcastle-Ottawa quality assessment scale**

| **Study** | **Selection score (max 5)** | **Comparability score (max 2)** | **Outcome score (max 3)** | **Total score (max 10)** | **Quality category** |
| --- | --- | --- | --- | --- | --- |
| Abramson 2017 [1] | 3 | 1 | 2 | 6 | Medium |
| Aceijas et al 2017 [2] | 5 | 2 | 2 | 9 | High |
| Açik et al 2019 [3] | 3 | 2 | 2 | 7 | Medium |
| Breiholz 2010 [4] | 1 | 1 | 1 | 3 | Low |
| Chacon-Cuberos et al. 2019 [5] | 5 | 1 | 1 | 7 | Medium |
| Chacon-Cuberos et al. 2018 [6] | 5 | 2 | 2 | 9 | High |
| El Ansari et al 2014 [7] | 3 | 1 | 2 | 6 | Medium |
| El Ansari et al 2015a [8] | 4 | 2 | 2 | 8 | High |
| El Ansari et al 2015b [9] | 3 | 2 | 2 | 7 | Medium |
| Fabian et al. 2013 [10] | 3 | 2 | 2 | 7 | Medium |
| Faghih et al 2020 [11] | 4 | 2 | 2 | 8 | High |
| Hamazaki et al. 2015 [12] | 4 | 2 | 2 | 8 | High |
| Hendy, 2012 [13] | 1 | 2 | 2 | 5 | Medium |
| Jaalouk et al 2019 [14] | 2 | 2 | 2 | 6 | Medium |
| Jeffers et al. 2019 [15] | 2 | 2 | 2 | 6 | Medium |
| Knowlden et al. 2016 [16] | 3 | 2 | 2 | 7 | Medium |
| Lesani et al., 2016 [17] | 3 | 2 | 2 | 7 | Medium |
| Liu et al. 2007 [18] | 4 | 2 | 2 | 8 | High |
| Lockhart, 2017 [19] | 3 | 2 | 2 | 7 | Medium |
| Lopez-Olivares et al. 2020 [20] | 4 | 2 | 2 | 8 | High |
| Lutz et al., 2017 [21] | 4 | 2 | 2 | 8 | High |
| Mikolajczyk et al., 2009 [22] | 3 | 2 | 2 | 7 | Medium |
| Mochimasu et al., 2016 [23] | 2 | 2 | 2 | 6 | Medium |
| Oleszko et al., 2019 [24] | 3 | 1 | 1 | 5 | Medium |
| Peltzer & Pengpid, 2017a [25] | 3 | 2 | 1 | 6 | Medium |
| Peltzer & Pengpid, 2017b [26] | 4 | 2 | 2 | 8 | High |
| Piqueras et al., 2011 [27] | 3 | 2 | 2 | 7 | Medium |
| Quehl et al 2017 [28] | 3 | 1 | 2 | 6 | Medium |
| Ramon Arbues et al 2019 [29] | 5 | 2 | 2 | 9 | High |
| Romijn 2020 [30] | 3 | 2 | 2 | 7 | Medium |
| Rossa-Roccor 2019 [31] | 3 | 2 | 2 | 7 | Medium |
| Sakai et al 2017 [32] | 4 | 2 | 2 | 8 | High |
| Schnettler et al., 2015 [33] | 3 | 2 | 2 | 7 | Medium |
| Smith-Marek et al., 2016 [34] | 3 | 2 | 2 | 7 | Medium |
| Tran et al., 2017 [35] | 3 | 2 | 2 | 7 | Medium |
| Wattick et al., 2018 [36] | 3 | 2 | 2 | 7 | Medium |
| Zurita-Ortegal et al., 2018 [37] | 3 | 2 | 2 | 7 | Medium |
| Rossa-Rocor et al., 2021 [38] | 4 | 2 | 2 | 8 | High |
| Stanton et al., 2021 [39] | 4 | 2 | 2 | 8 | High |
| Attlee et al., 2022 [40] | 4 | 2 | 2 | 8 | High |
| Lo Moro et al., 2021[41] | 3 | 1 | 2 | 6 | Medium |
| Lee et al., 2022 [42] | 3 | 2 | 2 | 7 | Medium |
| Alfreeh et al., 2020 [43] | 4 | 2 | 2 | 8 | High |
| Saharkhiz et al., 2021 [44] | 3 | 2 | 2 | 7 | Medium |

**Online supplementary material 2: Quality assessment (mental health effects on diet), based on Newcastle-Ottawa quality assessment scale**

| **Study** | **Selection score (max 5)** | **Comparability score (max 2)** | **Outcome score (max 3)** | **Total score (max 10)** | **Quality category** |
| --- | --- | --- | --- | --- | --- |
| Ahmed et al. 2014 [45] | 2 | 2 | 2 | 6 | Medium |
| Aljaber et al. 2019 [46] | 1 | 1 | 1 | 3 | Low |
| Almogbel et al. 2019 [47] | 2 | 1 | 2 | 5 | Medium |
| Bu et al. 2019 [48] | 2 | 1 | 2 | 5 | Medium |
| Cheng & Mohd Kamil, 2020 [49] | 3 | 1 | 1 | 5 | Medium |
| Leblanc & Villalon 2008 [50] | 2 | 1 | 1 | 4 | Medium |
| Dalton & Hammen 2018 [51] | 3 | 1 | 2 | 6 | Medium |
| El Ansari et al. 2015 [52] | 4 | 2 | 2 | 8 | High |
| Errisuriz et al., 2016 [53] | 3 | 2 | 2 | 7 | Medium |
| Hall et al., 2017 [54] | 3 | 2 | 2 | 7 | Medium |
| Kandiah et al., 2006 [55] | 3 | 2 | 1 | 6 | Medium |
| Keck et al., 2020 [56] | 4 | 2 | 2 | 8 | High |
| Kotecki et al. 2019 [57] | 2 | 1 | 1 | 4 | Medium |
| Lazarevich et al. 2018 [58] | 4 | 2 | 2 | 8 | High |
| Mansoury et al. 2015 [59] | 2 | 1 | 1 | 4 | Medium |
| Nataskin & Fiocco, 2015 [60] | 3 | 2 | 2 | 7 | Medium |
| Oliver & Wardle, 1999 [61] | 3 | 2 | 1 | 6 | Medium |
| Papier et al., 2015 [62] | 3 | 2 | 2 | 7 | Medium |
| Peker & Bermek, 2011 [63] | 3 | 2 | 2 | 7 | Medium |
| Pollard et al., 1995 [64] | 3 | 2 | 2 | 7 | Medium |
| Trigueros et al., 2020 [65] | 3 | 2 | 2 | 7 | Medium |
| Carlos et al., 2020 [66] | 3 | 1 | 2 | 6 | Medium |

**Online supplementary material 3: Quality assessment of review papers based on the guidance of the Centre for Reviews and Dissemination**

| **Study** | **Type of review** | **Aims** | **Population** | **Quality appraisal** |
| --- | --- | --- | --- | --- |
| Saha et al., 2021 [67] | Systematic review | To gather research evidence on the association between diet and depression and anxiety among college students. | University students (international) | Medium quality |
| Lyzwinski et al., 2018 [68] | Systematic review | To examine the patterns of dietary intake among university students experiencing stress, and to explore these relationships with weight. | University students (international) | Medium quality |

References

1	Abramson K. Understanding the Association Between Nutritionand Depression by Assessing Diet Quality,Omega-3, Cholesterol, and Sleep Quality inCollege Students . *Thesis And Dissertations* 2017;**648**.

2	Aceijas C, Waldhäusl S, Lambert N, *et al.* Determinants of health-related lifestyles among university students. *Perspect Public Health* 2017;**137**:227–236. doi:10.1177/1757913916666875

3	Açik M, Çakiroğlu FP. Evaluating the Relationship between Inflammatory Load of a Diet and Depression in Young Adults. *Ecol Food Nutr* 2019;**58**:366–378. doi:10.1080/03670244.2019.1602043

4	Breiholz R. *A correlation between diets higher in various proteins, fruits, and vegetables and depression scores*. 2010.

5	Chacón-Cuberos R, Zurita-Ortega F, Olmedo-Moreno EM, *et al.* Relationship between Academic Stress, Physical Activity and Diet in University Students of Education. *Behav Sci (Basel)* 2019;**9**. doi:10.3390/bs9060059

6	Chacón-Cuberos R, Zurita-Ortega F, Olmedo-Moreno EM, *et al.* An exploratory model of psychosocial factors and healthy habits in university students of physical education depending on gender. *Int J Environ Res Public Health* 2018;**15**. doi:10.3390/ijerph15112430

7	El Ansari W, Adetunji H, Oskrochi R. Food and mental health: relationship between food and perceived stress and depressive symptoms among university students in the United Kingdom. *Cent Eur J Public Health* 2014;**22**:90–97. doi:10.21101/cejph.a3941

8	El Ansari W, Suominen S, Berg-Beckhoff G. Mood and food at the University of Turku in Finland: nutritional correlates of perceived stress are most pronounced among overweight students. *Int J Public Health* 2015;**60**:707–716. doi:10.1007/s00038-015-0717-4

9	El Ansari W, Suominen S, Berg-Beckhoff G. Is Healthier Nutrition Behaviour Associated with Better Self-Reported Health and Less Health Complaints? Evidence from Turku, Finland. *Nutrients* 2015;**7**:8478–8490. doi:10.3390/nu7105409

10	Fabián C, Pagán I, Ríos JL, *et al.* Dietary patterns and their association with sociodemographic characteristics and perceived academic stress of college students in Puerto Rico. *P R Health Sci J* 2013;**32**:36–43.

11	Faghih S, Babajafari S, Mirzaei A, *et al.* Adherence to the dietary approaches to stop hypertension (DASH) dietary pattern and mental health in Iranian university students. *Eur J Nutr* 2020;**59**:1001–1011. doi:10.1007/s00394-019-01961-2

12	Hamazaki K, Natori T, Kurihara S, *et al.* Fish consumption and depressive symptoms in undergraduate students: A cross-sectional analysis. *Eur Psychiatry* 2015;**30**:983–987. doi:10.1016/j.eurpsy.2015.09.010

13	Hendy HM. Which comes first in food-mood relationships, foods or moods? *Appetite* 2012;**58**:771–775. doi:10.1016/j.appet.2011.11.014

14	Jaalouk D, Matar Boumosleh J, Helou L, *et al.* Dietary patterns, their covariates, and associations with severity of depressive symptoms among university students in Lebanon: a cross-sectional study. *Eur J Nutr* 2019;**58**:997–1008. doi:10.1007/s00394-018-1614-4

15	Jeffers AJ, Mason TB, Benotsch EG. Psychological eating factors, affect, and ecological momentary assessed diet quality. *Eat Weight Disord* Published Online First: 6 August 2019. doi:10.1007/s40519-019-00743-3

16	Knowlden AP, Hackman CL, Sharma M. Lifestyle and mental health correlates of psychological distress in college students. *Health Educ J* 2016;**75**:370–382. doi:10.1177/0017896915589421

17	Lesani A, Mohammadpoorasl A, Javadi M, *et al.* Eating breakfast, fruit and vegetable intake and their relation with happiness in college students. *Eat Weight Disord* 2016;**21**:645–651. doi:10.1007/s40519-016-0261-0

18	Liu C, Xie B, Chou C-P, *et al.* Perceived stress, depression and food consumption frequency in the college students of China Seven Cities. *Physiol Behav* 2007;**92**:748–754. doi:10.1016/j.physbeh.2007.05.068

19	Lockhart R. Predictive Relationship of Positive Lifestyle Choices with Emotional Distress of Undergraduate College Students: An Analysis of American College Health Association Data. *Predictive Relationship of Positive Lifestyle Choices with Emotional Distress of Undergraduate College Students: An Analysis of American College Health Association Data* 2017;:1–1.

20	López-Olivares M, Mohatar-Barba M, Fernández-Gómez E, *et al.* Mediterranean Diet and the Emotional Well-Being of Students of the Campus of Melilla (University of Granada). *Nutrients* 2020;**12**. doi:10.3390/nu12061826

21	Lutz LJ, Gaffney-Stomberg E, Williams KW, *et al.* Adherence to the Dietary Guidelines for Americans Is Associated with Psychological Resilience in Young Adults: A Cross-Sectional Study. *J Acad Nutr Diet* 2017;**117**:396–403. doi:10.1016/j.jand.2016.09.018

22	Mikolajczyk RT, El Ansari W, Maxwell AE. Food consumption frequency and perceived stress and depressive symptoms among students in three European countries. *Nutr J* 2009;**8**:31. doi:10.1186/1475-2891-8-31

23	Mochimasu KD, Miyatake N, Hase A. A pilot study of the relationship between diet and mental health in female university students enrolled in a training course for registered dietitians. *Environ Health Prev Med* 2016;**21**:345–349. doi:10.1007/s12199-016-0535-1

24	Oleszko A, Szczepańska E, Janion K, *et al.* Nutrition behaviours and the occurrence of depressive symptoms among the students in the institutions of higher education in Silesia (Poland). *Rocz Panstw Zakl Hig* 2019;**70**:69–77. doi:10.32394/rpzh.2019.0056

25	Peltzer K, Pengpid S. Dietary Behaviors, Psychological Well-Being, and Mental Distress Among University Students in ASEAN. *Iran J Psychiatry Behav Sci* 2017;**11**. doi:10.5812/ijpbs.10118

26	Peltzer K, Pengpid S. Dietary consumption and happiness and depression among university students: A cross-national survey. *Journal of Psychology in Africa* 2017;**27**:372–377. doi:10.1080/14330237.2017.1347761

27	Piqueras JA, Kuhne W, Vera-Villarroel P, *et al.* Happiness and health behaviours in Chilean college students: a cross-sectional survey. *BMC Public Health* 2011;**11**:443. doi:10.1186/1471-2458-11-443

28	Quehl R, Haines J, Lewis SP, *et al.* Food and Mood: Diet Quality is Inversely Associated with Depressive Symptoms in Female University Students. *Can J Diet Pract Res* 2017;**78**:124–128. doi:10.3148/cjdpr-2017-007

29	Ramón-Arbués E, Martínez Abadía B, Granada López JM, *et al.* [Eating behavior and relationships with stress, anxiety, depression and insomnia in university students.]. *Nutr Hosp* 2019;**36**:1339–1345. doi:10.20960/nh.02641

30	Romijn AR. Relationships among eating behaviour, diet quality, mental health and wellbeing in undergraduate students transitioning to university. *ISNPR* 2019.

31	Rossa-Roccor V. The association between diet and mental health and wellbeing in young adults within a biopsychosocial framework and a planetary health rationale. *University of British Columbia* Published Online First: 2019. doi:10.14288/1.0379616

32	Sakai H, Murakami K, Kobayashi S, *et al.* Food-based diet quality score in relation to depressive symptoms in young and middle-aged Japanese women. *Br J Nutr* 2017;**117**:1674–1681. doi:10.1017/S0007114517001581

33	Schnettler B, Miranda H, Lobos G, *et al.* Eating habits and subjective well-being. A typology of students in Chilean state universities. *Appetite* 2015;**89**:203–214. doi:10.1016/j.appet.2015.02.008

34	Smith-Marek EN, Durtschi J, Brown C, *et al.* Exercise and diet as potential moderators between trauma, posttraumatic stress, depression, and relationship quality among emerging adults. *Am J Fam Ther* 2016;**44**:53–66. doi:10.1080/01926187.2016.1145080

35	Tran A, Tran L, Geghre N, *et al.* Health assessment of French university students and risk factors associated with mental health disorders. *PLoS One* 2017;**12**:e0188187. doi:10.1371/journal.pone.0188187

36	Wattick RA, Hagedorn RL, Olfert MD. Relationship between Diet and Mental Health in a Young Adult Appalachian College Population. *Nutrients* 2018;**10**. doi:10.3390/nu10080957

37	Zurita-Ortega F, San Román-Mata S, Chacón-Cuberos R, *et al.* Adherence to the Mediterranean Diet Is Associated with Physical Activity, Self-Concept and Sociodemographic Factors in University Student. *Nutrients* 2018;**10**. doi:10.3390/nu10080966

38	Rossa-Roccor V, Richardson CG, Murphy RA, *et al.* The association between diet and mental health and wellbeing in young adults within a biopsychosocial framework. *PLoS One* 2021;**16**:e0252358. doi:10.1371/journal.pone.0252358

39	Stanton R, Best T, Williams S, *et al.* Associations between health behaviors and mental health in Australian nursing students. *Nurse Educ Pract* 2021;**53**:103084. doi:10.1016/j.nepr.2021.103084

40	Attlee A, Saravanan C, Shivappa N, *et al.* Higher Dietary Inflammatory Index Scores Are Associated With Stress and Anxiety in Dormitory-Residing Female University Students in the United Arab Emirates. *Front Nutr* 2022;**9**:814409. doi:10.3389/fnut.2022.814409

41	Lo Moro G, Corezzi M, Bert F, *et al.* Mental health and adherence to Mediterranean diet among university students: an Italian cross-sectional study. *J Am Coll Health* 2021;:1–11. doi:10.1080/07448481.2021.1970567

42	Lee CT, Ting GK, Bellissimo N, *et al.* The associations between lifestyle factors and mental well-being in baccalaureate nursing students: An observational study. *Nurs Health Sci* 2022;**24**:255–264. doi:10.1111/nhs.12923

43	Alfreeh L, Abulmeaty MMA, Abudawood M, *et al.* Association between the Inflammatory Potential of Diet and Stress among Female College Students. *Nutrients* 2020;**12**. doi:10.3390/nu12082389

44	Saharkhiz M, Khorasanchi Z, Karbasi S, *et al.* The association between adherence to a dietary approaches to stop hypertension (DASH) diet and neuro-psychological function in young women. *BMC Nutr* 2021;**7**:21. doi:10.1186/s40795-021-00429-z

45	Ahmed F, Al-Radhwan L, Al-Azmi G, *et al.* Association between Stress and Dietary Behaviours among Undergraduate Students in Kuwait Gender Differences. *Journal of Nutrition and Health Sciences* 2014;**1**. doi:10.15744/2393-9060.1.104

46	AlJaber MI, Alwehaibi AI, Algaeed HA, *et al.* Effect of academic stressors on eating habits among medical students in Riyadh, Saudi Arabia. *J Family Med Prim Care* 2019;**8**:390–400. doi:10.4103/jfmpc.jfmpc_455_18

47	Almogbel E, Aladhadh AM, Almotyri BH, *et al.* Stress associated alterations in dietary behaviours of undergraduate students of qassim university, saudi arabia. *Open Access Maced J Med Sci* 2019;**7**:2182–2188. doi:10.3889/oamjms.2019.571

48	Bu L, Lai Y, Deng Y, *et al.* Negative Mood Is Associated with Diet and Dietary Antioxidants in University Students During the Menstrual Cycle: A Cross-Sectional Study from Guangzhou, China. *Antioxidants (Basel)* 2019;**9**. doi:10.3390/antiox9010023

49	Cheng S-H, Mohd Kamil MK. Stress and Food Intake among University Students - Is There a Relationship? *JSM* 2020;**49**:121–128. doi:10.17576/jsm-2020-4901-14

50	Leblanc DD, Villalon L. [Perceived stress and its influence on eating behaviours of students at the University of Moncton]. *Canadian Journal of Dietetic Practice & Research*;**69**:133–140.

51	Dalton ED, Hammen CL. Independent and relative effects of stress, depressive symptoms, and affect on college students’ daily health behaviors. *J Behav Med* 2018;**41**:863–874. doi:10.1007/s10865-018-9945-4

52	El Ansari W, Berg-Beckhoff G. Nutritional Correlates of Perceived Stress among University Students in Egypt. *Int J Environ Res Public Health* 2015;**12**:14164–14176. doi:10.3390/ijerph121114164

53	Errisuriz VL, Pasch KE, Perry CL. Perceived stress and dietary choices: The moderating role of stress management. *Eat Behav* 2016;**22**:211–216. doi:10.1016/j.eatbeh.2016.06.008

54	Hall L, Tejada-Tayabas LM, Monárrez-Espino J. Breakfast Skipping, Anxiety, Exercise, and Soda Consumption are Associated with Diet Quality in Mexican College Students. *Ecol Food Nutr* 2017;**56**:218–237. doi:10.1080/03670244.2017.1299010

55	Kandiah J, Yake M, Jones J, *et al.* Stress influences appetite and comfort food preferences in college women. *Nutrition Research* 2006;**26**:118–123. doi:10.1016/j.nutres.2005.11.010

56	Keck MM, Vivier H, Cassisi JE, *et al.* Examining the Role of Anxiety and Depression in Dietary Choices among College Students. *Nutrients* 2020;**12**. doi:10.3390/nu12072061

57	Kotecki J, Kandiah J, Greene M, *et al.* Self-efficacy and Dietary Behaviors among Young Adults with Perceived Stress. *J Acad Nutr Diet* 2019;**119**:A74. doi:10.1016/j.jand.2019.06.216

58	Lazarevich I, Irigoyen Camacho ME, Velázquez-Alva MC, *et al.* Depression and food consumption in Mexican college students. *Nutr Hosp* 2018;**35**:620–626. doi:10.20960/nh.1500

59	Mansoury MM, McCullough F, Swift JA. The effect of academic stress on the dietary behaviour of female undergraduates in the Kingdom of Saudi Arabia. *Proc Nutr Soc* 2015;**74**. doi:10.1017/S0029665115004061

60	Nastaskin RS, Fiocco AJ. A survey of diet self-efficacy and food intake in students with high and low perceived stress. *Nutr J* 2015;**14**:42. doi:10.1186/s12937-015-0026-z

61	Oliver G, Wardle J. Perceived effects of stress on food choice. *Physiol Behav* 1999;**66**:511–515. doi:10.1016/s0031-9384(98)00322-9

62	Papier K, Ahmed F, Lee P, *et al.* Stress and dietary behaviour among first-year university students in Australia: sex differences. *Nutrition* 2015;**31**:324–330. doi:10.1016/j.nut.2014.08.004

63	Peker K, Bermek G. Predictors of health-promoting behaviors among freshman dental students at Istanbul University. *J Dent Educ* 2011;**75**:413–420. doi:10.1002/j.0022-0337.2011.75.3.tb05054.x

64	Pollard TM, Steptoe A, Canaan L, *et al.* Effects of academic examination stress on eating behavior and blood lipid levels. *Int J Behav Med* 1995;**2**:299–320. doi:10.1207/s15327558ijbm0204_2

65	Trigueros R, Padilla AM, Aguilar-Parra JM, *et al.* The Influence of Emotional Intelligence on Resilience, Test Anxiety, Academic Stress and the Mediterranean Diet. A Study with University Students. *Int J Environ Res Public Health* 2020;**17**. doi:10.3390/ijerph17062071

66	Carlos M, Elena B, Teresa IM. Are adherence to the mediterranean diet, emotional eating, alcohol intake, and anxiety related in university students in spain? *Nutrients* 2020;**12**. doi:10.3390/nu12082224

67	Saha S, Okafor H, Biediger-Friedman L, *et al.* Association between diet and symptoms of anxiety and depression in college students: A systematic review. *J Am Coll Health* 2021;:1–11. doi:10.1080/07448481.2021.1926267

68	Lyzwinski LN, Caffery L, Bambling M, *et al.* The Relationship Between Stress and Maladaptive Weight-Related Behaviors in College Students: A Review of the Literature. *American Journal of Health Education* 2018;**49**:166–178. doi:10.1080/19325037.2018.1449683
